# Navigating complexity: Evaluating management measures for multispecies snapper-grouper fisheries in the Southeast U.S. Atlantic

**DOI:** 10.1371/journal.pone.0355048

**Published:** 2026-08-14

**Authors:** Jie Cao, Kyle Shertzer, Scott Crosson, Erik Williams, Rick DeVictor

**Affiliations:** 1 Department of Applied Ecology, Center for Marine Sciences and Technology, North Carolina State University, Morehead City, North Carolina, United States of America; 2 Southeast Fisheries Science Center, Beaufort Laboratory, National Oceanic & Atmospheric Administration, National Marine Fisheries Service, Beaufort, North Carolina, United States of America; 3 Southeast Fisheries Science Center, Miami Laboratory, National Oceanic & Atmospheric Administration, National Marine Fisheries Service, Miami, Florida, United States of America; 4 Southeast Regional Office, National Oceanic & Atmospheric Administration, St. Petersburg, Florida, United States of America; Central Marine Fisheries Research Institute, INDIA

## Abstract

Multispecies fisheries are globally common, but typically managed through single-species controls. This situation can result in wasteful discarding, when fish of some species cannot be retained, but fishing effort toward the complex continues. In contrast, managing multispecies fisheries through effort controls can address this shortcoming. In this study, we used a simulation model to examine the effectiveness of various temporal and spatial effort controls applied to the multispecies snapper-grouper fishery in the southeast United States Atlantic. To represent the complex, we chose six species that are most commonly caught by the commercial and recreational sectors. We evaluated the various management scenarios based on performance metrics that quantify effects on spawning biomass, commercial and recreational landings, commercial and recreational discards, and economic indicators for both fishing sectors. These performance metrics were applied to both the species individually and in aggregate. We found that some management approaches were more effective than others, but that no single approach worked best for all species and both sectors. The optimal strategy depends on policy priorities, balancing potential tradeoffs between conservation goals (e.g., increased spawning biomass), harvest objectives (e.g., increased landings, decreased discards), and economic outcomes. Our modeling framework quantifies those tradeoffs.

## Introduction

In many marine systems worldwide, fishing effort can catch multiple species simultaneously or indiscriminately. In some cases, multispecies fisheries may target a single, desirable species but capture others as bycatch; in other cases, the fisheries target a complex of species that coexist on the same habitats. Either way, multispecies fisheries call for management approaches outside of traditional, single-species measures to avoid overfishing or stock collapse [1].

Fishery management approaches generally fall into one of two categories: output or input controls [2]. Output controls regulate what can be retained of the catch, utilizing such measures as size limits, bag or trip limits, or seasonal quotas. Input controls regulate fishing effort directly, typically by limiting access through spatial or temporal restrictions. A meaningful distinction is that output controls do not address fishing effort, but rather apply only after the effort has taken place. Thus, for multispecies fisheries with common effort, single-species output controls can result in the unintended consequence of substantial discarding [3]. This situation frustrates stakeholders, and for oceanic fishes with nonnegligible discard mortality rates, it wastes natural resources, forgoes yield, and can result in overfishing [4,5].

In the southeast United States (U.S.) Atlantic, commercial and recreational sectors target a multispecies complex of stocks often referred to as the snapper-grouper fishery. The fishery is managed by the South Atlantic Fishery Management Council (SAFMC) as part of their Snapper-Grouper Fishery Management Plan. This plan addresses management of 55 reef-associated finfishes, including snappers, groupers, porgies, jacks, tilefishes, and triggerfishes. The 55 species display a wide range of life histories, but coexist on shared hard-bottom habitat, such that fishing effort generally targets the complex rather than individual species. Most of these stocks have never been assessed.

To date, the complex has primarily been managed using single-species output controls. In this study, we explore the potential benefits of input controls and the tradeoffs that might occur among species. We focus on six species in a multispecies framework: black sea bass (*Centropristis striata*, [6]), gag grouper (*Mycteroperca microlepis*, [7]), red grouper (*Epinephelus morio*, [8]), red porgy (*Pagrus pagrus*, [9]), red snapper (*Lutjanus campechanus*, [10]), and vermilion snapper (*Rhomboplites aurorubens*, [11]). These species were chosen because of their importance to commercial and recreational sectors, and because they have stock assessments available to provide necessary information on life-history and fishery characteristics.

Here we use simulation analyses to navigate the complexity of applying input controls to manage the multispecies snapper-grouper fishery. We consider three types of input controls. The first reduces effort directly, which could be achieved through temporal openings/closures (e.g., [12–14]) or other means of limiting access (e.g., [15]). The second applies spatial regulations that limit access by region or depth (e.g., [14,16]). The third input control applies dynamic spatial regulations (e.g., [17]), similar to terrestrial crop rotations [18]. Our goal is to evaluate potential benefits of the various input controls to meet management objectives of healthy stocks, increased landings, decreased discarding, and economic benefits.

## Methods

A simulation-based approach, building on [19], was used to evaluate the performance of alternative management measures for the multispecies snapper-grouper fisheries in the southeast U.S. Atlantic. The simulation framework included a multi-species operating model (OM) that represents the population and fishery dynamics of the complex. The Methods section is organized as follows: we first describe the operating model, including its structure, parameterization, and conditioning. Next, we outline the management measures evaluated and detail how each was implemented within the simulation framework. Finally, we present the performance metrics used to compare the outcomes of the different management strategies.

**Fig 1:**
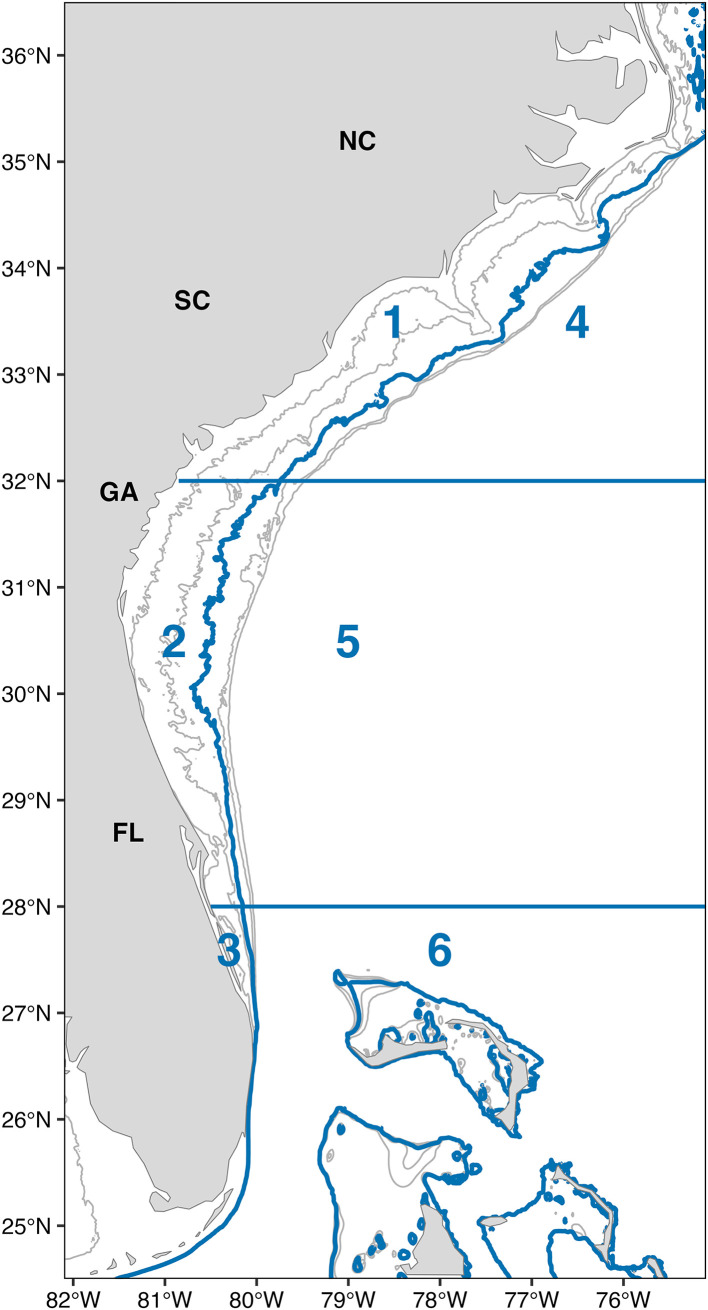
Study regions (1-6) across the southeast U.S. Atlantic coast. The area was divided into six model areas based on latitude and depth. Latitudinal boundaries at 28°N and 32°N divided the domain into northern, middle, and southern regions. Each region was further divided into nearshore and offshore areas using the 35-m isobath.

The framework was configured to simulate multispecies fisheries within the jurisdictional boundaries of the SAFMC, covering federally managed Atlantic waters off the southeast U.S. For this study, the SAFMC jurisdiction was subdivided into six spatial areas (Fig 1). Latitudinal boundaries at 28°N and 32°N delineated three regions: North Carolina and South Carolina (northern region), Georgia and northern Florida (middle region), and southern Florida (southern region). These regional divisions were designed to capture broad spatial patterns in both fish abundance and fishing effort. Each region was further split into nearshore and offshore zones, separated by the 35-meter isobath, to reflect depth-related variation in fish age-specific habitat use, fishing effort, and discard mortality.

### Operating model – Population dynamics

The OM tracks the abundance of multiple species simultaneously. For this study, we included red snapper, black sea bass, gag, red grouper, red porgy, and vermilion snapper – species that are commonly found in the region and play a key role in the snapper-grouper fishery. The abundance (*N*) of species *k* in area *i* at age *a* in year *y* is modeled using the exponential decay function:


$$\begin{array}{c} N_{k,i,a+\text{1},y+\text{1}}=N_{k,i,a,y}e^{-\left(F_{k,i,a,y}+M_{k,a}\right)} \end{array}$$ (1)

where $F_{k,i,a,y}$ is the fishing mortality rate, and $M_{k,a}$ is the natural mortality rate. We applied a maximum age of 50 years for all species to ensure that fewer than 0.5% of individuals would survive to the oldest age under natural mortality alone.

**Fig 2:**
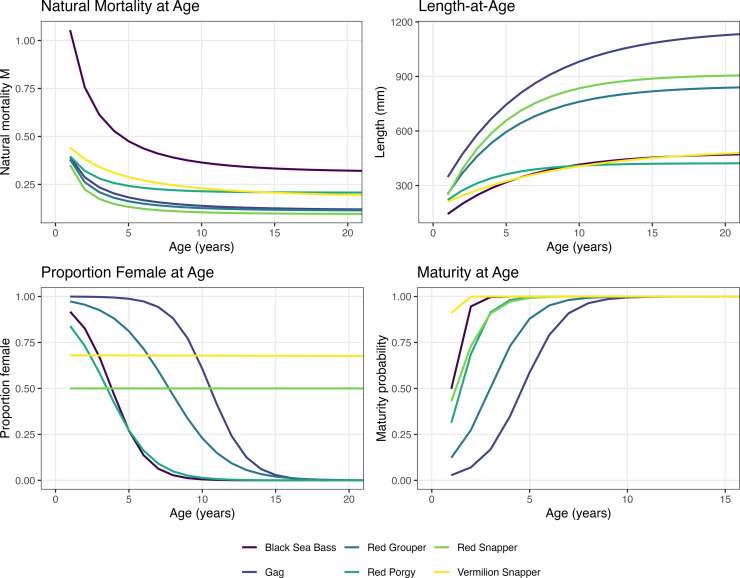
Natural mortality, length-at-age, proportion female, and maturity ogives for Red Snapper, Black Sea Bass, Gag, Red Grouper, Red Porgy, and Vermilion Snapper.

Natural mortality was assumed to follow an inverse length relationship, $M_{k,a}^{\prime }={L_{k,a}}^{-\text{1}}$, where $L_{k,a}$ is the length of species *k* at age *a*. This relationship assumes that natural mortality decreases as fish grow larger and has been widely used in marine fishes [20]. The age-specific natural mortality rate for each species, $M_{k,a}^{\prime }$, was then scaled to match the cumulative survival from age 2 onward that would be expected under the hypothetical age-invariant natural mortality rates used in the respective stock assessments (Table 1). These scaled vectors were used in the model as $M_{k,a}$ (Fig 2).

**Table 1:** Parameters used in the model.

Parameter	Description	Red snapper	Black sea bass	Gag	Red grouper	Red porgy	Vermilion snapper
$\gamma_{\text{1}}$	Weight-at-length coefficient	1.65E-8	5.02E-8	1.82E-8	8.418E-9	2.7E-5	2.1E-5
$\gamma_{\text{2}}$	Weight-at-length exponent	2.99	2.77	2.94	3.1	2.894	2.91
*K*	Growth coefficient	0.24	0.183	0.168	0.213	0.30	0.12
$L_{\infty }$	Asymptotic length	911	480	1161	848.2	422.6	506.0
$a_{\text{0}}$	Theoretical age at which length = 0	−0.33	−0.94	−1.11	−0.67	−1.47	−3.5
$R_{\text{0}}$	Unfished level of recruitment	4.37E5	7.08E7	5.27E5	3.60E5	3.4E6	6.3E6
h	Steepness of recruitment function	0.99	0.99	0.899	0.87	0.378	0.691
$\varphi_{\text{0}}$	Unfished spawners per recruit	0.017	9.07E-5	0.011	0.005	0.447	0.310
$\beta_{\text{1}}^{C}$	Fleet 1 catch selectivity slope	0.015	0.06	0.07	0.09	0.177	0.484
$\beta_{\text{2}}^{C}$	Fleet 2 catch selectivity slope	0.02	0.09	0.07	0.09	0.14	0.487
$\alpha_{\text{1}}^{C}$	Fleet 1 catch selectivity 50% location	343	217	200	289	254	261
$\alpha_{\text{2}}^{C}$	Fleet 2 catch selectivity 50% location	428	184	200	289	288	262
$\beta_{\text{1}}^{R}$	Fleet 1 retention slope	10	0.095	0.031	0.055	0.085	0.149
$\beta_{\text{2}}^{R}$	Fleet 2 retention slope	10	0.072	0.023	0.040	0.090	0.262
$\alpha_{\text{1}}^{R}$	Fleet 1 retention 50% location	0	345	591	493	344	275
$\alpha_{\text{2}}^{R}$	Fleet 2 retention 50% location	0	302	742	596	325	291
$\rho_{\text{1},i}$	Fleet 1 asymptotic retention in all areas *i*	0.15	1.0	1.0	1.0	1.0	1.0
$\rho_{\text{2},i}$	Fleet 2 asymptotic retention in all areas *i*	0.80	1.0	1.0	1.0	1.0	1.0
$\delta_{\text{1},i=\text{1},\text{2},\text{3}}$	Fleet 1 discard mortality rate near-shore	0.23	0.14	0.15	0.2	0.41	0.38
$\delta_{\text{1},i=\text{4},\text{5},\text{6}}$	Fleet 1 discard mortality rate off-shore	0.25	0.5	0.356	0.2	0.41	0.38
$\delta_{\text{2},i=\text{1},\text{2},\text{3}}$	Fleet 2 discard mortality rate near-shore	0.32	0.19	0.24	0.2	0.53	0.41
$\delta_{\text{2},i=\text{4},\text{5},\text{6}}$	Fleet 2 discard mortality rate off-shore	0.35	0.68	0.57	0.2	0.53	0.41
*q*_*1*_	Fleet 1 catchability	0.38	0.98	0.38	0.27	0.105	0.018
*q*_*2*_	Fleet 2 catchability	0.03	0.04	0.33	0.098	0.099	0.152
*M*	Natural mortality (invariant)	0.11	0.375	0.15	0.14	0.22	0.22
	Female maturity slope	1.28	2.87	0.975	0.987	1.57	4.52
	Female maturity 50% age	1.22	1.003	4.64	2.99	1.50	0.486
	Female proportion slope	0	−0.85	−0.79	−0.534	−0.66	0
	Female proportion 50% age	−10	3.83	10.55	7.73	3.51	−10

Recruits were defined as age-1 fish, and a Beverton-Holt stock-recruit relationship was assumed for each species. Specifically, recruitment to the system ($R_{k,y}$) was calculated as:


$$\begin{array}{c} R_{k,y}=\frac{\text{0}.\text{8}R_{k,\text{0}}h_{k}B_{k,y}}{\text{0}.\text{2}R_{k,\text{0}}\varphi_{k,\text{0}}\left(\text{1}-h_{k}\right)+B_{k,y}\left(h_{k}-\text{0}.\text{2}\right)} \end{array}$$ (2)

where $R_{k,\text{0}}$ is the unfished level of recruitment of species *k*, $h_{k}$ is the steepness parameter controlling the resilience of a stock, i.e., how sharply recruitment drops with declining spawning biomass, $\varphi_{k,\text{0}}$ is unfished spawners per recruit of species *k*, and $B_{k,y}$ is the annual spawning biomass of species *k*. For our study, steepness was set for each species according to the values used in their respective stock assessments (**Table 1**). For red snapper and black sea bass, steepness values are 0.99, approximating a null recruitment model in which recruitment is independent of spawning biomass [21]. In contrast, the other species included in the analysis exhibited a broader range of steepness values, from 0.9 to 0.3 (**Table 1**), reflecting varying degrees of density dependence in their stock-recruit relationships.

Spawning biomass ($B_{k,y}$) was measured as the total annual mature female biomass, summed across areas, for red snapper, black sea bass, and vermilion snapper. In contrast, for gag, red grouper, and red porgy, spawning biomass was measured as the total mature biomass, including both males and females. This difference between counting females only or both sexes in computation of spawning biomass matches the stock assessments, and it relates to gonochoristic versus protogynous life histories, a topic we return to in the Discussion. Within each area *i*, $B_{k,i,y}$ was computed as follows:


$$\begin{array}{c} B_{k,i,y}=\sum_{a}\left(p_{female,k}V_{a,k}+(\text{1}-p_{female,k})V_{male,k}\right)W_{a,k}N_{a,k,i,y} \end{array}$$ (3)

where $p_{female,k}$ is the proportion of females at age for species k (Fig 2). $V_{a,k}$ is the maturity at age vector for species k (Fig 2). Both proportion of females and maturity at age were modeled using a logistic function (Table 1). $V_{male,k}$ is a vector applied to male biomass – set to 0 for red snapper, black sea bass, and vermilion snapper (i.e., only female biomass contributes to spawning biomass) and to 1 for Gag, Red Grouper, and Red Porgy (i.e., all males are considered mature, and both male and female mature biomass are included). Weight at age $W_{a,k}$ followed a power function of length at age ($L_{a,k}$), $W_{a,k}=\gamma_{k,\text{1}}{L_{a,k}}^{\gamma_{k,\text{2}}}$, where $\gamma_{k,\text{1}}$ and $\gamma_{k,\text{2}}$ are parameters (Table 1). The term followed the standard von Bertalanffy growth function, $L_{a,k}=L_{\infty ,k}\left(\text{1}-e^{-K_{k}[a-a_{\text{0},k}]}\right)$, in which $L_{\infty ,k}$ is the maximum asymptotic length for species *k*, $K_{k}$ is the growth coefficient, and $a_{\text{0},k}$ is the theoretical age when length is zero (Fig 2).

The relative abundance of each area for each species was estimated by applying a multispecies configuration of the Vector Autoregressive Spatio-Temporal (VAST) package [22,23]). This model was applied to fishery-independent video survey data collected by the SouthEast Reef Fish Survey from 2011 to 2021. The model was fit simultaneously to video data for 21 species, including six focal species of this study. It modeled presence-absence and catch rates (number of individuals observed per video frame) as a multivariate process using latent spatial and spatiotemporal factors (for further details, see [24]. The VAST model produced abundance estimates on a 3- by 3-km grid. To estimate area-level relative abundance, we summed the predicted abundance within each of the six defined areas and then divided by the total abundance summed across all areas. This yielded $g_{k,i}$, the relative abundance of species *k* in area *i*, where $\sum_{i}g_{k,i}=\text{1}$ (Table 2).

**Table 2:** Proportion of Age-2 + fish allocated to each area by species.

Species	Area 1	Area 2	Area 3	Area 4	Area 5	Area 6
Red snapper	0.1132	0.6309	0.0118	0.0307	0.2066	0.0068
Black sea bass	0.4830	0.4476	0.0153	0.0208	0.0289	0.0044
Gag	0.3858	0.1805	0.0176	0.2302	0.1680	0.0180
Red grouper	0.2129	0.0789	0.0261	0.5044	0.1158	0.0619
Red porgy	0.2036	0.0171	0.0010	0.3414	0.4292	0.0077
Vermilion snapper	0.3239	0.2421	0.0111	0.1584	0.2611	0.0034

Allocation of recruits (age-1 fish) to areas varied by species based on their spatial life history characteristics. For red snapper, black sea bass, gag, and red grouper, recruits were distributed only across nearshore areas (i.e., Areas 1–3) in proportion to each region’s relative abundance (Table 3). In contrast, for red porgy and vermilion snapper, recruits were allocated across all six areas, with proportions matching the species’ relative abundance estimates (Table 3). Ontogenetic movement was modeled for age-2 fish – for each region, we imputed the proportion of age-2 fish that moved between nearshore area to the offshore area, such that equilibrium relative abundances in our model matched those provided by VAST (Table 2).

**Table 3:** Proportion of Age-1 fish allocated to each area by species.

Species	Area 1	Area 2	Area 3	Area 4	Area 5	Area 6
Red snapper	0.1439	0.8375	0.0186	0.0000	0.0000	0.0000
Black sea bass	0.5038	0.4765	0.0197	0.0000	0.0000	0.0000
Gag	0.6160	0.3484	0.0356	0.0000	0.0000	0.0000
Red grouper	0.7173	0.1947	0.0880	0.0000	0.0000	0.0000
Red porgy	0.3239	0.2421	0.0111	0.1584	0.2611	0.0034
Vermilion snapper	0.2036	0.0171	0.0010	0.3414	0.4292	0.0077

### Operating model – Fishery dynamics

Total fishing mortality was modeled as the sum of mortality contributions from two fleets, each consisting of both a landings and discards component. Fleet 1 (f = 1) represented recreational fishing in Atlantic waters off the southeast U.S., while the Fleet 2 (f = 2) represented commercial fishing. For each fleet, we modeled selectivity at age of the catch for each species *k* at each area *i* in a given year *y* as a logistic function of length as follows:


$$\begin{array}{c} S_{f,k,i,a,y}^{C}=\frac{\text{1}}{\left[\text{1}+e^{-\beta_{f,k,i,y}^{C}\left(L_{k,a}-\alpha_{f,k,i,y}^{C}\right)}\right]} \end{array}$$ (4)

Where $\beta_{f,k,i,y}^{C}$ defines the slope and $\alpha_{f,k,i,y}^{C}$ defines the length at 50% selection. The catch was apportioned between landings and discards according to a retention ogive ($S_{f,k,i,a,y}^{R}$) as follows:


$$\begin{array}{c} S_{f,k,i,a,y}^{R}=\frac{\rho_{f,k,i,y}}{\left[\text{1}+e^{-\beta_{f,k,i,y}^{R}\left(L_{k,a}-\alpha_{f,k,i,y}^{R}\right)}\right]} \end{array}$$ (5)

where $\beta_{f,k,i,y}^{R}$ defines the slope, $\alpha_{f,k,i,y}^{R}$ defines the length at 50% retention, and $\rho_{f,k,i,y}$ defines the asymptotic retention that can range between 0 (all fish discarded) and 1 (maximum possible retention). With logistic catch selectivity, a value of $\rho_{f,k,i,y}<\text{1}$ models the situation where discards include the larger fish in the population.

Given the catch selectivity and retention function, selectivity of landings ($S_{f,k,i,t,a}^{L}$) is the product $S_{f,k,i,a,y}^{L}=S_{f,k,i,a,y}^{C}S_{f,k,i,a,y}^{R}$ and selectivity of discards is the product $S_{f,k,i,a,y}^{D}=S_{f,k,i,a,y}^{C}\left({\text{1}-S}_{f,k,i,a,y}^{R}\right)$. For each fleet, the age-specific fishing mortality rate is


$$\begin{array}{c} F_{f,k,i,a,y}=\left(S_{f,k,i,a,y}^{L}+\delta_{f,k,i}S_{f,k,i,a,y}^{D}\right)\Phi_{f,k,i,y} \end{array}$$ (6)

where $\delta_{f,k,i}$ is the fleet- and area-specific discard mortality rate for species *k*, and $\Phi_{f,k,i,y}$ is the total fishing mortality rate of fleet *f* in area *i* and year *y* for species *k*. The total fishing mortality rate (equation 1) in each area for each species as $F_{k,i,a,y}=\sum_{f}F_{f,k,i,a,y}$

We computed the fishing mortality term $\Phi_{f,k,i,y}=q_{f,k,i,y}E_{f,k,i,y}$ where $q_{f,k,i,y}$ is catchability coefficient and $E_{f,k,i,y}$ is fishing effort, both of which are management foci of this study. Effort was assumed to be shared across species within each fleet, as the focal species in this study form a multispecies complex in that they occupy overlapping habitats and are often captured together.

Following the approach of Shertzer et al. [19], we defined a base level of effort for each fleet as $E_{f}=\text{1}$, such that any spatiotemporal variation in effort reflects relative change. For example, a 50% reduction in effort in area *i* would be modeled as $E_{f,i}=\text{0}.\text{5}$. The spatial apportionment of fishing effort among areas followed the configuration used by Shertzer et al. [19].

Fleet-specific base catchability values ($q_{f}$) were derived assuming $E_{f}=\text{1}$ and calibrated using fishing mortality rates from the most recent stock assessments, averaged over the stable management period of 2010–2019. These base values could be modified by area or year to simulate the effects of spatial or temporal variation in catchability due to management measures. Importantly, the $q_{f}$ values are not direct measures of gear efficiency but rather represent relative fishing mortality rates associated with the recreational and commercial fleets.

Discard mortality rates were modeled separately for recreational and commercial fisheries, with values differentiated between nearshore and offshore areas to account for spatial variation in survival rates (Table 1). Species-specific discard mortality rates were assigned based on the stock assessments and available literature, reflecting differences in depth, handling practices, and physiological tolerance among species. In general, discard mortality rates were assumed to be higher in offshore areas where deeper depths result in more stress and barotrauma [25].

Given the mortality rates, landings (*L*) and dead discards (*D*) can be calculated using the Baranov catch equation (Baranov 1918; Sharov 2021):


$$\begin{array}{c} L_{f,k,i,a,y}=\frac{{S_{f,k,i,a,y}^{L}\Phi }_{f,k,i,y}}{F_{k,i,a,y}+M_{k,a}}N_{k,i,a,y}\left(\text{1}-e^{-\left(F_{k,i,a,y}+M_{k,a}\right)}\right) \end{array}$$ (7)

$$\begin{array}{c} D_{f,k,i,a,y}=\frac{{\delta_{f,k,i}S_{f,k,i,a,y}^{D}\Phi }_{f,k,i,y}}{F_{k,i,a,y}+M_{k,a}}N_{k,i,a,y}\left(\text{1}-e^{-\left(F_{k,i,a,y}+M_{k,a}\right)}\right) \end{array}$$ (8)

Then the total landings and discards by fleet for each species were calculated by summing those values by areas and year.

### Management scenarios design

The management scenarios considered in this study encompassed a range of input-control strategies, including temporal, spatial, and spatiotemporal measures, aimed at evaluating how alternative configurations of fishing effort and retention affect population, fishery, and economic outcomes. We evaluated 16 scenarios using the multispecies operating model (Table 4). These scenarios were designed as structured management experiments to isolate the effects of different management mechanism. Although some of these measures could be implemented jointly in practice, we evaluated them individually to isolate their effects and better understand the potential contributions of each toward achieving management objectives.

**Table 4:** Management scenarios evaluated in the multispecies operating model.

Scenario^*^	Scenario group	Description
s1	System-wide effort reduction	All effort reduced by 25%
s2		All effort reduced by 75%
s3	Recreational effort reduction	Recreational effort reduced by 25%
s4		Recreational effort reduced by 75%
s5	Recreational effort reduction + retention bounding case	Recreational effort reduced by 25%, full retention
s6		Recreational effort reduced by 75%, full retention
s7	Depth-based spatial closure	Close offshore areas to effort, efforts shift to nearshore
s8	Regional spatial closures, all fleets	Close northern region to all effort
s9		Close middle region to all effort
s10		Close southern region to all effort
s11	Depth-based recreational closure	Close offshore areas to recreational effort, efforts shift to nearshore
s12	Regional recreational closures	Close northern region to Recreational effort
s13		Close middle region to Recreational effort
s14		Close southern region to Recreational effort
s15 s16	Dynamic spatial management	Rotating nearshore and offshore openings every other year Rotating regional openings, such that each region is closed every third year

We organized the scenarios into five scenario families. The first family represented system-wide effort reductions, in which fishing effort for both fleets was reduced by either 25% or 75% across all areas (s1–s2). These scenarios provide broad benchmarks for the expected effects of reducing total fishing mortality on the multispecies complex. The second family represented recreational effort reductions, in which effort was reduced only for Fleet 1, the recreational sector, by either 25% or 75% (s3–s4). These scenarios isolate the effects of reducing recreational fishing mortality while maintaining commercial effort at baseline levels.

The third family combined recreational effort reductions with a hypothetical full-retention assumption (s5–s6). In the model, full retention was implemented by setting the asymptotic retention parameter, $\rho_{f,k,i,y}$, to 1.0 for the recreational fleet after the management change. Retention remained length-dependent through the existing retention ogive, so this change should be interpreted as removing the upper cap on retention rather than forcing retention of every encountered fish. These scenarios shift a portion of catch from the discard component to the landing component and therefore represent hypothetical bounding cases for evaluating the potential consequences of eliminating regulatory discards.

The fourth family represented fixed spatial closures. These included closures of offshore areas, with effort redirected to nearshore areas (s7), closures of northern, middle, or southern regions to both fleets (s8–s10), and analogous closures applied only to the recreational fleet (s11–s14). When offshore areas were closed, fishing effort was assumed to be redirected to the corresponding nearshore areas rather than eliminated, reflecting a simplified behavioral response by fishers. For other regional closures, effort outside the closed region was maintained at baseline levels, and no additional behavioral redistribution was modeled.

The fifth family represented dynamic spatial management. Scenario s15 rotated nearshore and offshore openings every other year, whereas s16 rotated regional openings so that each latitudinal region was closed every third year. These scenarios were included to evaluate whether rotating access could provide a compromise between permanent closures and unrestricted access.

### Sensitivity analyses and performance metrics

To evaluate the effects of alternative management measures, we conducted deterministic simulations using the multispecies operating model to estimate equilibrium outcomes. Our objective was to compare expected long-term outcomes across management mechanisms under specific assumptions. We first ran the model under base-level conditions until it reached equilibrium. We then ran each management scenario by modifying the relevant effort or retention parameters beginning in year 101 and allowing the system to approach a new equilibrium. Scenario outcomes were summarized by comparing values at year 200 with the base-level equilibrium at year 100. This two-phase structure allowed comparisons between status quo and managed conditions after both had reached equilibrium.

For biological and fishery outcomes, we computed five metrics for each species and scenario: spawning biomass, recreational landings, commercial landings, recreational dead discards, and commercial dead discards. Landings and dead discards were computed in numbers of fish. For these metrics, we report percent change relative to base-level conditions. A value of 100% indicates that the metric doubled relative to status quo, whereas a value of −100% indicates complete elimination of the metric.

To assess the economic implications, we evaluated both commercial and recreational outcomes using the same valuation metrics used by NOAA Fisheries Service Southeast Regional Office (Table 5). For the commercial sector, we estimated gross ex-vessel revenue using 5-year average from the logbook data and producer surplus [26,27]), representing landings values minus variable costs (including the opportunity costs of labor). For the recreational sector, we applied consumer surplus values per fish for both retained and discarded catches [28]. All values were expressed in 2023 U.S. dollars, adjusted using the GDP deflator. These four metrics provide a standardized basis for comparing the economic tradeoffs of alternative management strategies across fleets and species.

**Table 5:** Economic values associated with commercial and recreational fisheries for six species. Commercial values are reported in dollars per pound, while recreational values are per fish, all adjusted to 2023 dollars using the GDP deflator from the Bureau of Economic Analysis.

Species	Commercial gross revenue	Commercial producer surplus	Recreational retained consumer surplus	Recreational discarded consumer surplus
Red snapper	6.93	2.61	100	11
Black sea bass	3.82	1.14	128	16
Red grouper	6.33	1.98	128	16
Gag grouper	7.79	2.43	128	16
Vermillion snapper	4.79	1.52	100	11
Red porgy	2.92	1.1	100	11

Because management priorities may differ among biological conservation, harvest, discard reduction, and economic objectives, we did not use a single aggregate score as the primary basis for identifying a preferred scenario. Instead, we present tradeoffs among metrics and scenario families. As a supplementary analysis, we calculated illustrative scenario scores under alternative weighting schemes to show how scenario rankings depend on management priorities. These scores were calculated after scaling each biological/fishery metric across scenarios, reversing the sign of discard metrics so that lower dead discards corresponded to higher performance. The resulting scores are intended only as sensitivity analyses of weighting assumptions.

To better understand the effects of effort reduction intensity on both the biological and economic performance of the fishery, we also conducted a sensitivity analysis across a range of effort reduction levels, from 10% to 90% in 5% increments. We examined three policy variants: (i) reducing recreational effort with full retention, (ii) reducing effort in both recreational and commercial sectors with full retention, and (iii) reducing recreational effort without full retention. These analyses provide a higher-resolution examination of the temporal effort-reduction scenarios and clarify how biological and economic outcomes respond to the magnitude of effort reduction.

All simulations and analyses were performed using R version 4.5.0 (R Core Team 2022).

## Results

### Biological and fisheries tradeoffs across management scenarios

Across the 16 scenarios, management outcomes varied strongly among biological and fishery metrics, and no scenario simultaneously increased spawning biomass, increased landings, and reduced dead discards in both sectors. Instead, scenario performance depended on the management mechanism being evaluated and on whether the priority was conservation, harvest, or discard reduction.

**Fig 3:**
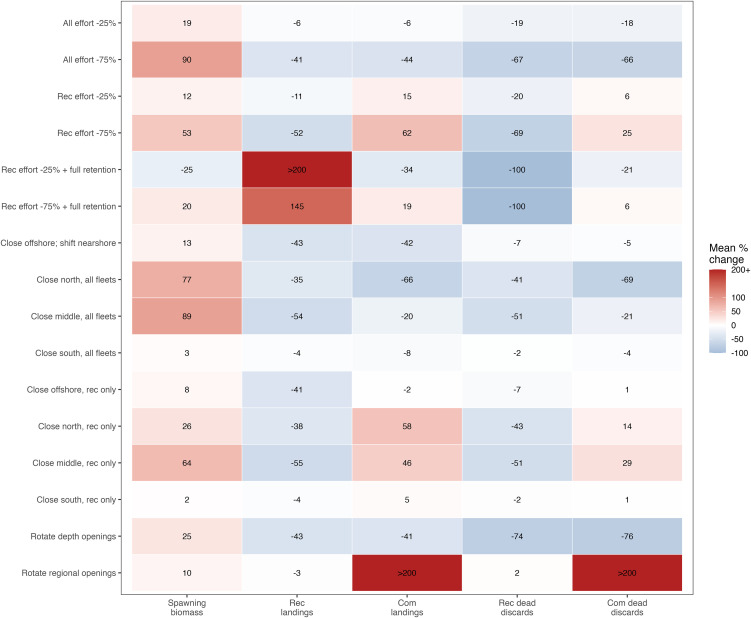
Mean percent change across the six focal species in spawning biomass, recreational landings, commercial landings, recreational dead discards, and commercial dead discards under each management scenario. Values are relative to status quo equilibrium conditions. Colors indicate the direction and magnitude of change, with blue representing decreases and orange representing increases. The color scale is capped at −100% and +200%; cells labeled “>200” exceeded the upper plotting limit. For spawning biomass and landings, positive values indicate increases. For dead discards, negative values indicate reductions in discard mortality.

Mean responses across the six focal species showed several broad patterns (Fig 3). System-wide effort reductions increased spawning biomass and reduced dead discards, but reduced landings in both sectors. The stronger system-wide effort reduction scenario, s2, produced a larger mean increase in spawning biomass than s1, but also produced larger reductions in recreational and commercial landings. Recreational-only effort reductions showed a different sectoral pattern: they increased spawning biomass and reduced recreational landings and recreational dead discards, while commercial landings generally increased because commercial effort remained at baseline. This pattern was especially clear under s4, which reduced recreational effort by 75% and produced a mean spawning biomass increase while also increasing commercial landings.

The hypothetical full-retention scenarios produced the strongest contrast between retained recreational catch and other objectives. Scenario s5, which combined a 25% reduction in recreational effort with hypothetical full retention, produced a large mean increase in recreational landings but a mean decline in spawning biomass. Scenario s6, which combined a 75% reduction in recreational effort with hypothetical full retention, also increased recreational landings but produced a positive mean spawning biomass response. These results indicate that full retention can substantially increase retained catch in the model, but the biological consequences depend on whether the increase in retention is offset by sufficient reductions in effort.

Fixed spatial closures produced heterogeneous outcomes. Closing the northern or middle region to all fleets produced relatively large mean increases in spawning biomass and reductions in dead discards, but also reduced landings. In contrast, closing the southern region produced relatively small mean changes across most metrics. Recreational-only regional closures tended to increase spawning biomass and commercial landings while reducing recreational landings, reflecting the reduction in recreational fishing mortality while commercial effort remained unchanged. Dynamic spatial scenarios also differed strongly from one another. Rotating depth openings reduced landings and dead discards in both sectors, whereas rotating regional openings produced a large increase in commercial landings and commercial dead discards with little mean change in recreational landings. These contrasting responses demonstrate that spatial and dynamic spatial measures redistribute both benefits and costs among sectors and metrics rather than uniformly improving all outcomes.

### Species-specific biological and fishery responses

**Fig 4:**
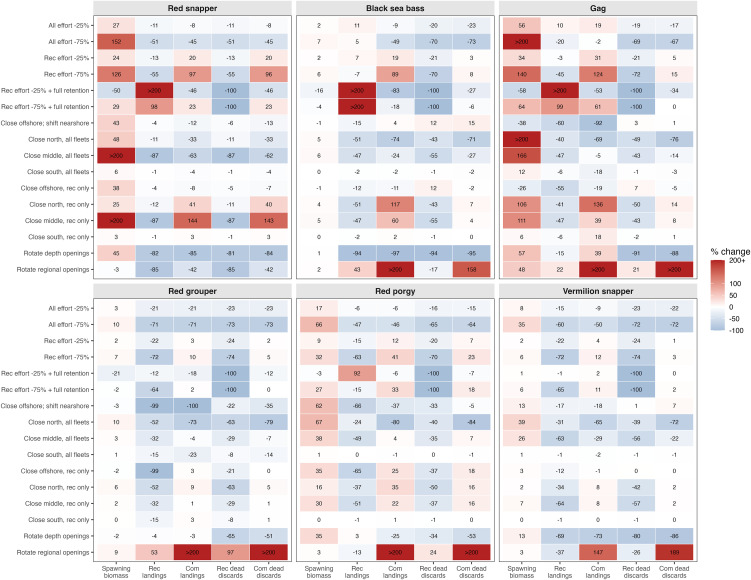
Percent change in spawning biomass, recreational landings, commercial landings, recreational dead discards, and commercial dead discards for each focal species under each management scenario. Values are relative to status quo equilibrium conditions. Colors indicate the direction and magnitude of change, with blue representing decreases and orange representing increases. The color scale is capped at −100% and +200%; cells labeled “>200” exceeded the upper plotting limit. For spawning biomass and landings, positive values indicate increases. For dead discards, negative values indicate reductions in discard mortality.

Species-specific responses revealed that average responses across the complex masked substantial variation among species (Fig 4). The same management scenario often produced different outcomes across species, reflecting differences in life history, spatial distribution, catchability, selectivity, retention, and discard mortality. For example, effort reduction and regional closure scenarios generally produced stronger spawning biomass responses for gag, red porgy, and red snapper than for some other species. In contrast, black sea bass showed particularly large changes in recreational landings under the full-retention scenarios, reflecting the interaction between high modeled recreational catch and the change in retention assumptions.

The full-retention scenarios showed the clearest species-level contrast. Scenario s5 increased recreational landings for several species, but this did not consistently translate into improved biomass outcomes. For some species, particularly red snapper, modest recreational effort reduction combined with increased retention produced weaker or negative biomass responses. Scenario s6, with stronger recreational effort reduction, produced more favorable biomass outcomes while still increasing recreational landings for some species. These results emphasize that retention-based outcomes cannot be interpreted independently from effort levels and species-specific population responses.

### Economic valuation

**Fig 5:**
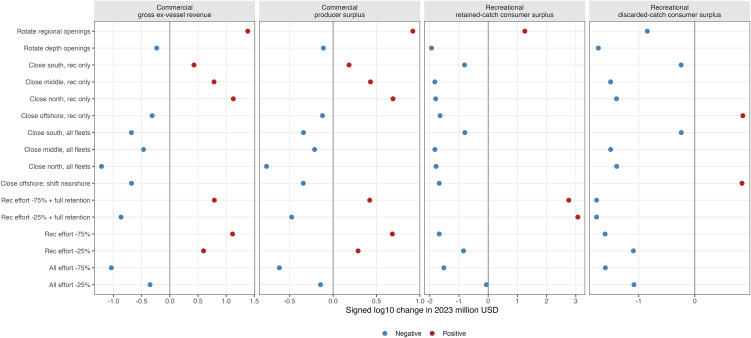
Total change in economic value across the six focal species for commercial gross ex-vessel revenue, commercial producer surplus, recreational retained-catch consumer surplus, and recreational discarded-catch consumer surplus. Values are relative to status quo equilibrium conditions and expressed in 2023 U.S. dollars. The x-axis uses a signed log10 transformation of values in million U.S. dollars to preserve positive and negative changes while compressing large magnitudes. Blue points indicate negative economic changes and orange points indicate positive economic changes. The vertical line indicates no change relative to status quo.

Economic outcomes also varied strongly among scenarios and sectors. Summed across the six focal species, commercial gross ex-vessel revenue and commercial producer surplus generally increased under scenarios that reduced recreational effort while maintaining commercial effort, including s4 and several recreational-only spatial closures (Fig 5). This pattern reflects the biological benefit of reduced recreational fishing mortality combined with continued commercial access. Conversely, system-wide effort reductions and many all-fleet spatial closures reduced commercial economic outcomes because commercial fishing effort or access was also reduced.

Recreational retained-catch consumer surplus showed a different pattern. The largest positive responses occurred under the hypothetical full-retention scenarios, especially s5 and s6. These gains were driven primarily by increased retained recreational catch, particularly for black sea bass, under the assumption that a greater portion of selected catch could be retained.

Recreational discarded-catch consumer surplus generally declined under scenarios that reduced recreational effort or shifted catch from the discard component to the retained component. In some spatial scenarios, discarded-catch value increased slightly, reflecting scenario-specific changes in the number of released fish. Overall, economic results reinforced the biological and fishery tradeoffs: scenarios that improved commercial economic outcomes often reduced recreational outcomes, while scenarios that produced large recreational retained-catch values depended strongly on the full-retention assumption.

**Fig 6:**
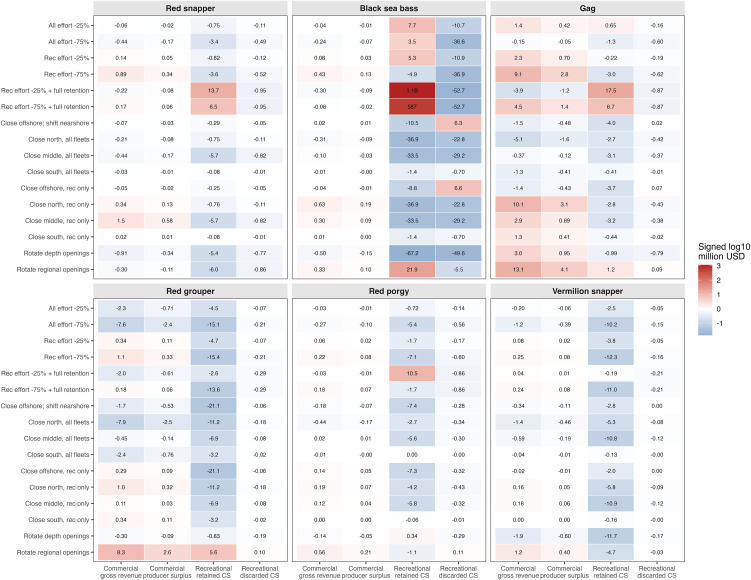
Change in economic value for each focal species under each management scenario, shown for commercial gross revenue, commercial producer surplus, recreational retained-catch consumer surplus, and recreational discarded-catch consumer surplus. Cell values are changes relative to status quo equilibrium conditions in 2023 million U.S. dollars; “B” denotes billion U.S. dollars. Colors show signed log10-transformed values in million U.S. dollars, with blue indicating negative changes and orange indicating positive changes.

Species-level economic responses further illustrated the source of these patterns (Fig 6). The largest species-specific economic values occurred for black sea bass recreational retained-catch consumer surplus under the full-retention scenarios, whereas commercial economic gains were distributed more broadly across scenarios that reduced recreational effort or altered spatial access.

### Sensitivity analysis of effort reduction

The effort-reduction sensitivity analysis showed that biological, fishery, and economic outcomes changed nonlinearly as effort was reduced from 10% to 90% of baseline. These sensitivity runs provide a higher-resolution view of the temporal effort-reduction scenarios and help clarify how the magnitude of effort reduction affected tradeoffs among spawning biomass, landings, dead discards, and economic value.

**Fig 7:**
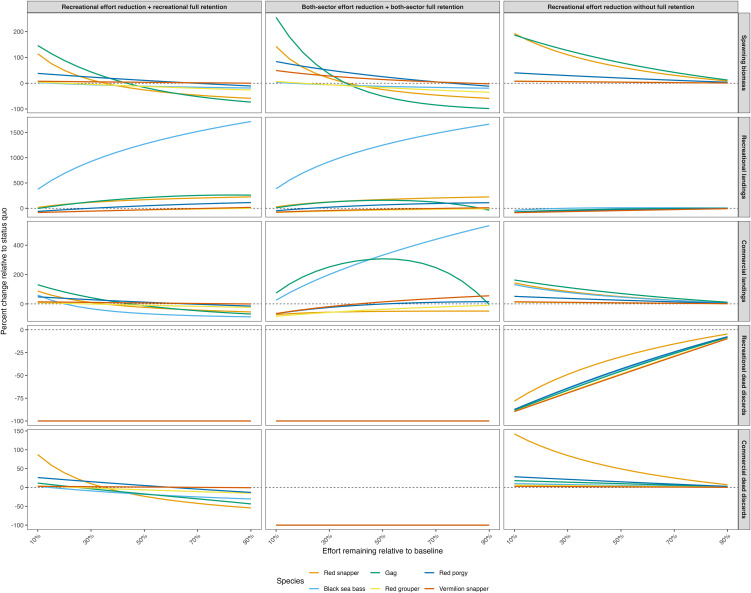
Percent change in equilibrium spawning biomass, recreational landings, commercial landings, recreational dead discards, and commercial dead discards relative to status quo as effort remaining varied from 90% to 10% of baseline. Lines show species-specific responses for the six focal species. Columns represent three sensitivity variants: recreational effort reduction with recreational full retention, both-sector effort reduction with both-sector full retention, and recreational effort reduction without full retention. The horizontal dashed line indicates no change relative to status quo. Positive values indicate increases in spawning biomass, landings, or dead discards; negative values indicate decreases. For dead discards, negative values represent reductions in discard mortality.

For spawning biomass, effort reductions had the strongest positive effects for gag and red snapper, followed by red porgy, whereas responses were more muted for vermilion snapper, black sea bass, and red grouper (Fig 7). This pattern is consistent with the scenario results, where effort-reduction and regional-closure scenarios produced stronger biomass responses for some species than others. Recreational dead discards generally declined as recreational effort decreased, particularly in the sensitivity scenario without full retention. However, commercial dead discards can increase nonlinearly as recreational effort declined. Landings generally declined as effort was reduced, although gag showed a dome-shaped response in which landings initially increased as effort was reduced to approximately 50% of baseline and then declined with further effort reduction (Fig 7).

**Fig 8:**
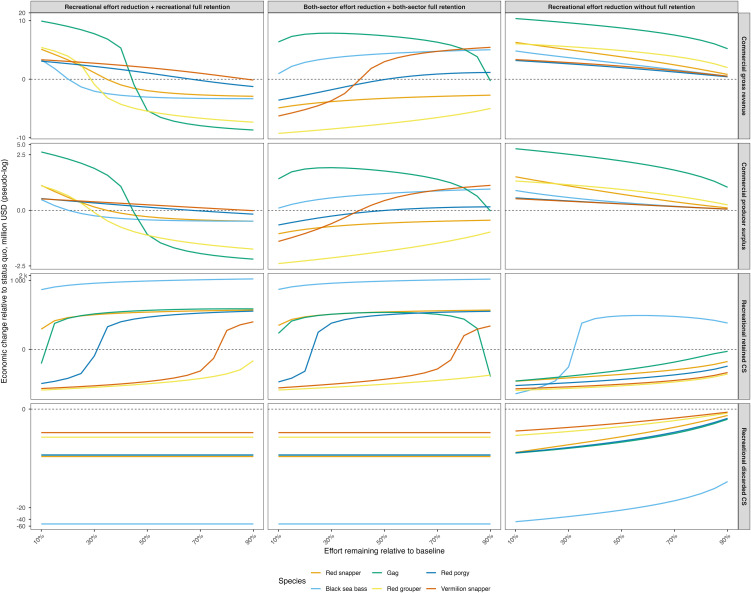
Species-specific changes in commercial gross revenue, commercial producer surplus, recreational retained-catch consumer surplus, and recreational discarded-catch consumer surplus relative to status quo as effort remaining varied from 90% to 10% of baseline. Values are expressed in 2023 U.S. dollars and plotted on a pseudo-log scale to display both positive and negative economic changes across a wide range of magnitudes. Lines represent individual species. Columns represent recreational effort reduction with recreational full retention, both-sector effort reduction with both-sector full retention, and recreational effort reduction without full retention. The horizontal dashed line indicates no change relative to status quo.

Economic responses to effort reduction also varied by species, sector, and retention assumption (Fig 8). Without full retention, commercial economic metrics generally increased as recreational effort decreased, whereas recreational economic metrics declined. Black sea bass was an exception: recreational retained-catch consumer surplus remained relatively stable until effort declined to approximately 35% of baseline, after which it decreased sharply. Under full-retention assumptions, several species showed nonlinear economic responses. For example, commercial gross revenue showed a reverse S-shaped relationship with effort for gag and red grouper when full retention was applied in the recreational sector, whereas recreational retained-catch consumer surplus showed an S-shaped response for red porgy and vermilion snapper under recreational-only full retention (Fig 8). These nonlinear patterns indicate that economic responses cannot be inferred simply from proportional changes in effort.

**Fig 9:**
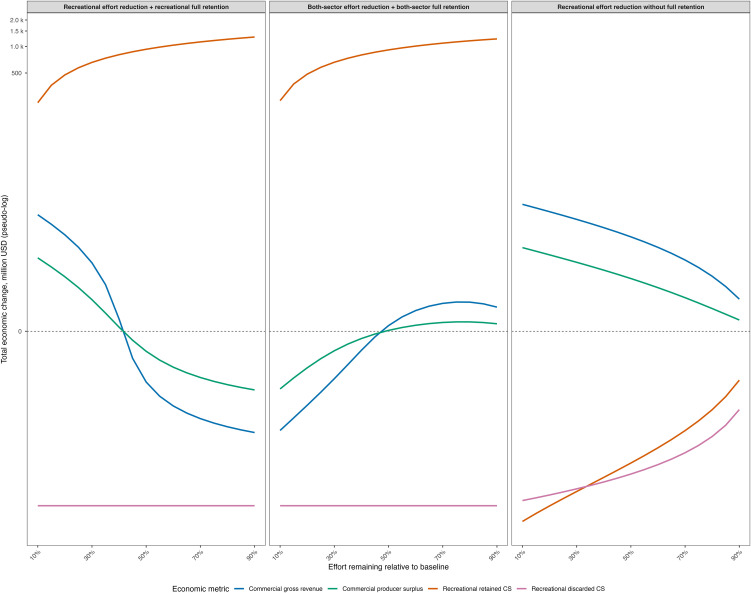
Total changes in economic metrics summed across the six focal species as effort remaining varied from 90% to 10% of baseline. Lines show commercial gross revenue, commercial producer surplus, recreational retained-catch consumer surplus, and recreational discarded-catch consumer surplus. Values are expressed in 2023 U.S. dollars and plotted on a pseudo-log scale to preserve positive and negative values while compressing large absolute changes. Columns represent recreational effort reduction with recreational full retention, both-sector effort reduction with both-sector full retention, and recreational effort reduction without full retention. The horizontal dashed line indicates no change relative to status quo.

Aggregated across species, the three sensitivity variants produced distinct sectoral patterns (Fig 9). In the scenario without full retention, commercial economic metrics increased as recreational effort decreased, while recreational economic metrics declined. When full retention was applied only to the recreational sector, commercial metrics showed a nonlinear response with an inflection near intermediate effort levels. When full retention was applied to both sectors, commercial metrics showed a different nonlinear response, with the strongest changes occurring near intermediate effort reductions.

Overall, the sensitivity analysis supports two conclusions. First, the magnitude of effort reduction matters: moderate and strong effort reductions can produce qualitatively different outcomes, especially when retention assumptions change. Second, responses are often nonlinear and species-specific, so management effects cannot be summarized by a simple proportional relationship between effort and outcome.

## Discussion

The management of multispecies fisheries is a complex challenge, as traditional single-species approaches often fail to account for species interactions and shared fishing effort. Our study used a simulation framework to evaluate the potential of various temporal and spatial input controls to manage the U.S. Atlantic snapper-grouper complex. The findings highlight the tradeoffs between conservation goals (e.g., increased spawning biomass), harvest objectives (e.g., increased landings, decreased discards), and economic outcomes.

Across the 16 scenarios, no management strategy simultaneously increased spawning biomass, increased landings, reduced dead discards, and improved economic outcomes for all species and both sectors. Broad effort reductions and some fixed spatial closures generally increased spawning biomass and reduced dead discards, but often reduced landings. Recreational-only effort reductions tended to increase spawning biomass and commercial outcomes while reducing recreational landings. Hypothetical full-retention scenarios generated large increases in recreational retained catch and associated consumer surplus, but these gains depended on the level of effort reduction and did not always align with biomass improvements. Dynamic spatial scenarios also produced contrasting outcomes: rotating depth openings tended to reduce landings and discards, whereas rotating regional openings increased commercial landings and economic value but also increased commercial dead discards. These patterns demonstrate that scenario performance depends on the management objective being emphasized.

This result reinforces the need to evaluate multispecies management strategies as tradeoffs rather than as a single ranked list of alternatives. A scenario that appears favorable when averaged across species may not perform well for each individual species, and a scenario that improves outcomes for one sector may impose costs on another. For example, recreational-only effort reductions can benefit spawning biomass and commercial landings because commercial effort remains at baseline while recreational fishing mortality declines. However, that same mechanism reduces recreational landings and may increase commercial discards for some species. Similarly, rotating regional openings can increase commercial landings but may also increase commercial dead discards. These results highlight the importance of explicitly identifying management priorities before comparing scenarios.

**Fig 10:**
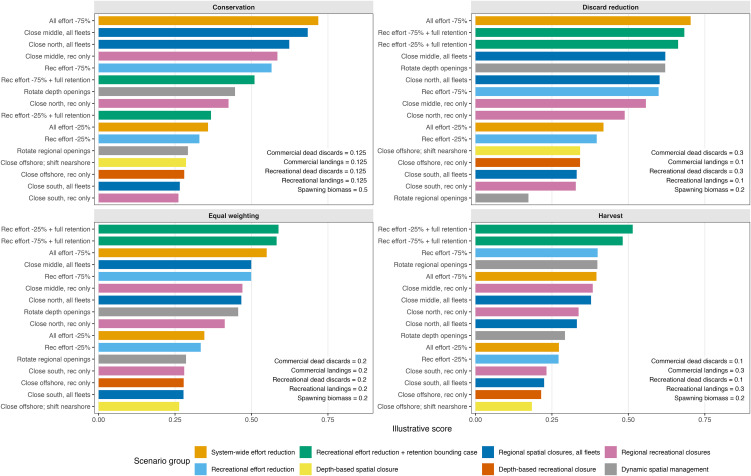
Illustrative scenario scores calculated under four weighting schemes: conservation, discard reduction, harvest, and equal weighting. Scores were based on biological and fishery metrics: spawning biomass, recreational landings, commercial landings, recreational dead discards, and commercial dead discards. For discard metrics, lower dead discards were treated as higher performance. The weights assigned to each metric are shown within each panel. Bar colors indicate scenario families.

The sensitivity of scenario interpretation to management priorities was further illustrated by the alternative weighting analysis (Fig 10). When spawning biomass was weighted most heavily, the highest-scoring scenarios were strong system-wide effort reduction, closure of the middle region to all fleets, and closure of the northern region to all fleets. In contrast, when discard reduction was emphasized, strong effort reduction and full-retention bounding scenarios ranked more highly, along with some fixed regional closures and rotating depth openings. Harvest-weighted scores favored scenarios that maintained or increased landings, including the full-retention bounding scenarios, recreational effort reduction, and rotating regional openings. Equal weighting produced yet another ordering, with full-retention scenarios ranking highly because of their strong effects on recreational landings and recreational dead discards. These differences show that scenario rankings are not inherent properties of the model outputs; rather, they depend on the weights assigned to biomass, landings, and discards.

Species-specific responses were also important. Gag, red porgy, and red snapper were generally more responsive to effort reductions and regional closures than some other species, whereas black sea bass showed especially large changes in recreational landings and recreational retained-catch value under the hypothetical full-retention scenarios. For gag and red porgy, strong spawning biomass responses may partly reflect their protogynous life histories and the way spawning biomass was represented in the simulations. In the model, spawning biomass for gag, red grouper, and red porgy included both mature males and females, matching the corresponding stock assessment conventions. For protogynous species, fishing mortality can disproportionately remove larger, older males, potentially disrupting spawning dynamics [29,30]). Effort reductions or spatial closures that protect larger fish can therefore strengthen the spawning stock. Black sea bass is also protogynous, but spawning biomass was represented as mature female biomass to match the stock assessment, which may partly explain why its biomass response differed from gag and red porgy. These differences emphasize that multispecies management evaluations need to account for species-specific life histories.

Spatial management remains a potentially useful tool, but our results suggest that its effects depend strongly on where closures occur and which fleets are affected. Closing the northern or middle region to all fleets generally increased spawning biomass and reduced dead discards, but at the cost of reduced landings. Closing the southern region produced smaller changes, suggesting that spatial closures are not interchangeable. Their effects depend on the overlap among species distributions, fishing effort, selectivity, and discard mortality. Recreational-only spatial closures often shifted benefits toward commercial outcomes while reducing recreational landings. These results suggest that spatial management may be useful for targeting particular biological or sectoral objectives, but that the design of spatial measures would need to be closely tied to species distributions and fleet behavior.

Dynamic spatial management may provide a way to distribute access restrictions through space and time, but the two rotating scenarios produced different tradeoffs. Rotating nearshore and offshore openings reduced landings and dead discards in both sectors, whereas rotating regional openings increased commercial landings and commercial value but also increased commercial dead discards. These results suggest that rotating access can redistribute fishing opportunities and biological impacts, but it does not eliminate tradeoffs. The effectiveness of this approach is likely tied to the spatial distribution of fishing effort and fish populations. By closing an area with a high concentration of fishing activity, the stocks can grow and provide spillover benefits to surrounding regions, as with Marine Protected Areas (MPAs) [31,32]. Of course, closing large areas indefinitely is unpopular among stakeholders. Rotating the closures/openings may be considered more equitable, and regional rotations (s16) tended to increase landings and economic benefit, along with modest increase to spawning biomass. To make spatial scenarios more palatable, we can envision a system of potentially rotating, large-area closures, but with small-area openings within. For example, within the closed area, artificial reefs and some designated natural reefs could remain open to provide fishing opportunities. In essence, this approach would invert the concept of MPAs, by providing pockets of Marine Exploitable Areas. These MEAs could potentially be highly productive fishing grounds, if they receive appreciable spillover from the surrounding, protected areas.

As with any modeling study, results should be interpreted in light of underlying assumptions. We evaluated expected (asymptotic) results from deterministic simulations to compare across management scenarios. However, transient short-term effects may also be of interest to managers. Simulations designed to examine transient dynamics would benefit from including stochasticity in several model features, such as recruitment, fishing effort, and fish movement. In addition, we did not attempt to model any effort re-allocation, other than the full shift of effort from offshore to inshore when offshore was closed. For other spatial scenarios, we assumed no effort reallocation, and for temporal scenarios, we assumed that the reduction already included any potential re-allocation. In real applications, managers could anticipate that at least some effort re-allocation might occur. Finally, the model did not include ecological interactions among species, habitat-mediated dynamics, or climate-driven distribution shifts, all of which may be relevant for reef-associated multispecies fisheries. Species interactions such as competition, predation, or shared density dependence could affect population responses, and habitat structure or habitat-specific productivity could influence the effects of spatial management. Future extensions could incorporate reef structure, habitat covariates, species co-occurrence, or interaction terms to evaluate whether habitat-mediated and ecological processes alter the projected effects of spatial management scenarios.

Ideally, the economic valuation of the recreational sector would include the marginal tradeoffs between the number of trips cancelled or restricted and the increased retained catch on remaining trips, rather than the linear valuations applied here. Currently, such data do not exist but would be a logical next step for evaluating various criteria in a real-life application. Because our model produced estimates of biomass and total catch rather than effort, per-fish landings valuations were required and are congruent with NOAA’s current cost-benefit methodologies for proposed regulatory changes in this region. NOAA does not currently use the Liese and Carter (2012) valuations for discarded catch, but we included them here to illustrate the roughly nine-to-one increase in value for retained versus discarded fish in this complex of species.

Currently, the private recreational fleet of this multispecies fishery is managed as open access. This situation is not likely sustainable, given the heavy and increasing recreational effort [33], paired with ever-increasing fishing power [34], applied to relatively small hardbottom reefs with well-advertised locations comprising a small percentage of the seafloor [35]. Rather than single-species output controls, managing effort, especially in a multispecies fishery, can bring economic, ecological, and angling-quality benefits [36,37]). Thus, our study addressed managing through input controls, with several scenarios focused on recreational fishing effort. The intent behind those scenarios was that restrictions to effort would apply to the private recreational fleet, noting that the commercial fleet is already managed as limited entry through a permit system, and for-hire components of the recreational fleet (headboats, charterboats) could be managed similarly to maintain their year-round business model.

Our model provides a framework for evaluating the complex tradeoffs associated with managing a multispecies fishery using input controls. The choice of an optimal strategy depends on the specific priorities of managers and stakeholders. Future work to inform management of this multispecies snapper-grouper fishery could build on our modeling framework and incorporate priorities through management strategy evaluation [38]. Such an endeavor will need to navigate the complexity of balancing potentially competing priorities of building spawning biomass, harvest potential, minimizing discards, and economic stability across sectors and across species. Our study is designed to support a move in that direction.

## References

[1] Burgess MG, Polasky S, Tilman D. Predicting overfishing and extinction threats in multispecies fisheries. Proc Natl Acad Sci U S A. 2013;110(40):15943–8. doi: 10.1073/pnas.1314472110 24043810 PMC3791778

[2] Bellido JM, Sumaila UR, Sánchez-Lizaso JL, Palomares ML, Pauly D. Input versus output controls as instruments for fisheries management with a focus on Mediterranean fisheries. Marine Policy. 2020;118:103786. doi: 10.1016/j.marpol.2019.103786

[3] Browne M, Calderwood J, Brophy D, Minto C. Single-species quotas drive discards by multi-species trawlers in the Celtic Seas ecoregion when their relative abundance fluctuates. ICES Journal of Marine Science. 2024;81(9):1745–63. doi: 10.1093/icesjms/fsae122

[4] Abbott JK, Wilen JE. Regulation of fisheries bycatch with common-pool output quotas. Journal of Environmental Economics and Management. 2009;57(2):195–204. doi: 10.1016/j.jeem.2008.04.003

[5] Harrington JM, Myers RA, Rosenberg AA. Wasted fishery resources: discarded by‐catch in the USA. Fish and Fisheries. 2005;6(4):350–61. doi: 10.1111/j.1467-2979.2005.00201.x

[6] SEDAR. SEDAR 76 South Atlantic Black Sea Bass Stock Assessment Report. North Charleston SC: SEDAR. 2023. https://sedarweb.org/assessments/sedar-76/

[7] SEDAR. SEDAR 71 South Atlantic Gag Stock Assessment Report. North Charleston SC: SEDAR. 2021a. http://sedarweb.org/sedar-71

[8] SEDAR. SEDAR 53 – South Atlantic Red Grouper Assessment Report. North Charleston, SC: SEDAR. 2017. http://sedarweb.org/sedar-53

[9] SEDAR. SEDAR 60 South Atlantic Red Porgy Stock Assessment Report. 2020. SEDAR, North Charleston SC. 181 http://sedarweb.org/sedar-60

[10] SEDAR. 2021b. SEDAR 73 South Atlantic Red Snapper Stock Assessment Report. 2021b. SEDAR, North Charleston SC. 194 http://sedarweb.org/sedar-73

[11] SEDAR. SEDAR 55 – South Atlantic Vermilion Snapper Assessment Report. North Charleston, SC: SEDAR. 2018. http://sedarweb.org/sedar-55

[12] Chagaris D, Allen M, Camp E. Modeling temporal closures in a multispecies recreational fishery reveals tradeoffs associated with species seasonality and angler effort dynamics. Fisheries Research. 2019;210:106–20. doi: 10.1016/j.fishres.2018.10.018

[13] Hyman AC, Chagaris D, Drexler M, Frazer TK. Modeling effort in a multispecies recreational fishery; Influence of species-specific temporal closures, relative abundance, and seasonality on monthly angler-trips. Fisheries Research. 2024;279:107136. doi: 10.1016/j.fishres.2024.107136

[14] Little LR, Punt AE, Mapstone BD, Begg GA, Goldman B, Ellis N. Different responses to area closures and effort controls for sedentary and migratory harvested species in a multispecies coral reef linefishery. ICES Journal of Marine Science. 2009;66(9):1931–41. doi: 10.1093/icesjms/fsp164

[15] Johnston RJ, Holland DS, Maharaj V, Campson TW. Fish harvest tags: An alternative management approach for recreational fisheries in the US Gulf of Mexico. Marine Policy. 2007;31(4):505–16. doi: 10.1016/j.marpol.2006.12.004

[16] Holland DS. Integrating spatial management measures into traditional fishery management systems: the case of the Georges Bank multispecies groundfish fishery. ICES Journal of Marine Science. 2003;60(5):915–29. doi: 10.1016/s1054-3139(03)00097-3

[17] Poulton AJ, Sethi SA, Ellner SP, Smeltz TS. Optimal dynamic spatial closures can improve fishery yield and reduce fishing-induced habitat damage. Can J Fish Aquat Sci. 2023;80(6):893–912. doi: 10.1139/cjfas-2022-0198

[18] Plagányi ÉE, Skewes T, Murphy N, Pascual R, Fischer M. Crop rotations in the sea: Increasing returns and reducing risk of collapse in sea cucumber fisheries. Proc Natl Acad Sci U S A. 2015;112(21):6760–5. doi: 10.1073/pnas.1406689112 25964357 PMC4450382

[19] Shertzer K, Crosson S, Williams E, Cao J, DeVictor R, Dumas C, et al. Fishery management strategies for Red Snapper in the southeastern U.S. Atlantic: A spatial population model to compare approaches. North American Journal of Fisheries Management. 2024;44(1):113–31. doi: 10.1002/nafm.10966

[20] Lorenzen K. Size- and age-dependent natural mortality in fish populations: Biology, models, implications, and a generalized length-inverse mortality paradigm. Fisheries Research. 2022;255:106454. doi: 10.1016/j.fishres.2022.106454

[21] Brooks EN. Pragmatic approaches to modeling recruitment in fisheries stock assessment: A perspective. Fisheries Research. 2024;270:106896. doi: 10.1016/j.fishres.2023.106896

[22] Thorson JT. Guidance for decisions using the Vector Autoregressive Spatio-Temporal (VAST) package in stock, ecosystem, habitat and climate assessments. Fisheries Research. 2019;210:143–61. doi: 10.1016/j.fishres.2018.10.013

[23] Thorson JT, Barnett LAK. Comparing estimates of abundance trends and distribution shifts using single- and multispecies models of fishes and biogenic habitat. ICES Journal of Marine Science. 2017;74(5):1311–21. doi: 10.1093/icesjms/fsw193

[24] Cao J, Craig JK, Damiano MD. Spatiotemporal dynamics of Atlantic reef fishes off the southeastern U.S. coast. Ecosphere. 2024;15(6). doi: 10.1002/ecs2.4868

[25] Pulver JR. Sink or swim? Factors affecting immediate discard mortality for the gulf of Mexico commercial reef fish fishery. Fisheries Research. 2017;188:166–72. doi: 10.1016/j.fishres.2016.12.018

[26] Liese C, Crosson S. Quantifying the economic effects of different fishery management regimes in two otherwise similar fisheries. PLoS One. 2023;18(6):e0287250. doi: 10.1371/journal.pone.0287250 37339153 PMC10281562

[27] Liese C. Economics of the U.S. South Atlantic Snapper-Grouper Fishery - 2018. NOAA. 2023.

[28] Carter DW, Liese C. The Economic Value of Catching and Keeping or Releasing Saltwater Sport Fish in the Southeast USA. North American Journal of Fisheries Management. 2012;32(4):613–25. doi: 10.1080/02755947.2012.675943

[29] Coleman FC, Koenig CC, Collins LA. Reproductive styles of shallow-water groupers (Pisces: Serranidae) in the eastern Gulf of Mexico and the consequences of fishing spawning aggregations. Environ Biol Fish. 1996;47(2):129–41. doi: 10.1007/bf00005035

[30] Huntsman GR, Potts J, Mays RW, Vaughan D. Groupers (Serranidae, Epinephelinae): Endangered apex predators of reef communities. In: Musick JA. Life in the slow lane: Ecology and conservation of long-lived marine animals. American Fisheries Society. 1999. p. 217–31.

[31] Bode M, Choukroun S, Emslie MJ, Harrison HB, Leis JM, Mason LB, et al. Marine reserves contribute half of the larval supply to a coral reef fishery. Sci Adv. 2025;11(6):eadt0216. doi: 10.1126/sciadv.adt0216 39908375 PMC11797529

[32] Sala E, Giakoumi S. No-take marine reserves are the most effective protected areas in the ocean. ICES Journal of Marine Science. 2017;75(3):1166–8. doi: 10.1093/icesjms/fsx059

[33] Shertzer KW, Williams EH, Craig JK, Fitzpatrick EE, Klibansky N, Siegfried KI. Recreational sector is the dominant source of fishing mortality for oceanic fishes in the Southeast United States Atlantic Ocean. Fisheries Management Eco. 2019;26(6):621–9. doi: 10.1111/fme.12371

[34] Cooke SJ, Venturelli P, Twardek WM, Lennox RJ, Brownscombe JW, Skov C, et al. Technological innovations in the recreational fishing sector: implications for fisheries management and policy. Rev Fish Biol Fish. 2021;31(2):253–88. doi: 10.1007/s11160-021-09643-1 33642705 PMC7900803

[35] Steward DN, Paxton AB, Bacheler NM, Schobernd CM, Mille K, Renchen J, et al. Quantifying spatial extents of artificial versus natural reefs in the seascape. Front Mar Sci. 2022;9. doi: 10.3389/fmars.2022.980384

[36] Waters JR. Restricted access vs. open access methods of management: Toward a more effective regulation of fishing effort. Marine Fisheries Review. 1991;53:1–10.

[37] Cox S, Walters C. Maintaining Quality in Recreational Fisheries: How Success Breeds Failure in Management of Open‐Access Sport Fisheries. Recreational Fisheries. Wiley. 2002. 107–19. doi: 10.1002/9780470995402.ch8

[38] Punt AE, Butterworth DS, de Moor CL, De Oliveira JAA, Haddon M. Management strategy evaluation: best practices. Fish and Fisheries. 2014;17(2):303–34. doi: 10.1111/faf.12104

