# Supplementary material for: Navigating complexity: Evaluating management measures for multispecies snapper-grouper fisheries in the Southeast U.S. Atlantic
Source: PLoS One. 2026 Aug 14;21(8):e0355048. doi: 10.1371/journal.pone.0355048 (PMC13475873; doi:10.1371/journal.pone.0355048)
Supplement: Supplementary file 1 [file pone.0355048.s002.docx]

PONE-D-26-09475

Navigating Complexity: Evaluating Management Measures for Multispecies Snapper-Grouper Fisheries in the Southeast U.S. Atlantic

PLOS One

Dear Dr. Cao,

Thank you for submitting your manuscript to PLOS ONE. After careful consideration, we feel that it has merit but does not fully meet PLOS ONE’s publication criteria as it currently stands. Therefore, we invite you to submit a revised version of the manuscript that addresses the points raised during the review process.

Please submit your revised manuscript by May 20 2026 11:59PM. If you will need more time than this to complete your revisions, please reply to this message or contact the journal office at [plosone@plos.org](mailto:plosone@plos.org). When you're ready to submit your revision, log on to https://www.editorialmanager.com/pone/ and select the 'Submissions Needing Revision' folder to locate your manuscript file.

Please include the following items when submitting your revised manuscript:

- A letter that responds to each point raised by the academic editor and reviewer(s). You should upload this letter as a separate file labeled 'Response to Reviewers'.
- A marked-up copy of your manuscript that highlights changes made to the original version. You should upload this as a separate file labeled 'Revised Manuscript with Track Changes'.
- An unmarked version of your revised paper without tracked changes. You should upload this as a separate file labeled 'Manuscript'.

If you would like to make changes to your financial disclosure, please include your updated statement in your cover letter. Guidelines for resubmitting your figure files are available below the reviewer comments at the end of this letter.

If applicable, we recommend that you deposit your laboratory protocols in protocols.io to enhance the reproducibility of your results. Protocols.io assigns your protocol its own identifier (DOI) so that it can be cited independently in the future. For instructions see: <https://journals.plos.org/plosone/s/submission-guidelines#loc-laboratory-protocols>. Additionally, PLOS ONE offers an option for publishing peer-reviewed Lab Protocol articles, which describe protocols hosted on protocols.io. Read more information on sharing protocols at <https://plos.org/protocols?utm_medium=editorial-email&utm_source=authorletters&utm_campaign=protocols>.

As the corresponding author, your ORCID iD is verified in the submission system and will appear in the published article. PLOS supports the use of ORCID, and we encourage all coauthors to register for an ORCID iD and use it as well. Please encourage your coauthors to verify their ORCID iD within the submission system before final acceptance, as unverified ORCID iDs will not appear in the published article. *Only* the individual author can complete the verification step; PLOS staff *cannot* verify ORCID iDs on behalf of authors.

We look forward to receiving your revised manuscript.

Kind regards,

Abdul Azeez Pokkathappada, Ph.D.
Academic Editor
PLOS One

**Journal Requirements:**

When submitting your revision, we need you to address these additional requirements.

1. Please ensure that your manuscript meets PLOS ONE's style requirements, including those for file naming. The PLOS ONE style templates can be found at

<https://journals.plos.org/plosone/s/file?id=wjVg/PLOSOne_formatting_sample_main_body.pdf>and

<https://journals.plos.org/plosone/s/file?id=ba62/PLOSOne_formatting_sample_title_authors_affiliations.pdf>

2. Please note that PLOS One has specific guidelines on code sharing for submissions in which author-generated code underpins the findings in the manuscript. In these cases, we expect all author-generated code to be made available without restrictions upon publication of the work. Please review our guidelines at https://journals.plos.org/plosone/s/materials-and-software-sharing#loc-sharing-code and ensure that your code is shared in a way that follows best practice and facilitates reproducibility and reuse.

3. Please note that your Data Availability Statement is currently missing the DOI/accession number of each dataset OR a direct link to access each database. If your manuscript is accepted for publication, you will be asked to provide these details on a very short timeline. We therefore suggest that you provide this information now, though we will not hold up the peer review process if you are unable.

4. We note that Figure 1 in your submission contain map images which may be copyrighted. All PLOS content is published under the Creative Commons Attribution License (CC BY 4.0), which means that the manuscript, images, and Supporting Information files will be freely available online, and any third party is permitted to access, download, copy, distribute, and use these materials in any way, even commercially, with proper attribution. For these reasons, we cannot publish previously copyrighted maps or satellite images created using proprietary data, such as Google software (Google Maps, Street View, and Earth). For more information, see our copyright guidelines: http://journals.plos.org/plosone/s/licenses-and-copyright.

We require you to either (1) present written permission from the copyright holder to publish these figures specifically under the CC BY 4.0 license, or (2) remove the figures from your submission:

1. You may seek permission from the original copyright holder of Figure 1 to publish the content specifically under the CC BY 4.0 license.

We recommend that you contact the original copyright holder with the Content Permission Form (http://journals.plos.org/plosone/s/file?id=7c09/content-permission-form.pdf) and the following text:

“I request permission for the open-access journal PLOS ONE to publish XXX under the Creative Commons Attribution License (CCAL) CC BY 4.0 (http://creativecommons.org/licenses/by/4.0/). Please be aware that this license allows unrestricted use and distribution, even commercially, by third parties. Please reply and provide explicit written permission to publish XXX under a CC BY license and complete the attached form.”

Please upload the completed Content Permission Form or other proof of granted permissions as an "Other" file with your submission.

In the figure caption of the copyrighted figure, please include the following text: “Reprinted from [ref] under a CC BY license, with permission from [name of publisher], original copyright [original copyright year].”

2. If you are unable to obtain permission from the original copyright holder to publish these figures under the CC BY 4.0 license or if the copyright holder’s requirements are incompatible with the CC BY 4.0 license, please either i) remove the figure or ii) supply a replacement figure that complies with the CC BY 4.0 license. Please check copyright information on all replacement figures and update the figure caption with source information. If applicable, please specify in the figure caption text when a figure is similar but not identical to the original image and is therefore for illustrative purposes only.

The following resources for replacing copyrighted map figures may be helpful:

USGS National Map Viewer (public domain): http://viewer.nationalmap.gov/viewer/

The Gateway to Astronaut Photography of Earth (public domain): http://eol.jsc.nasa.gov/sseop/clickmap/

Maps at the CIA (public domain): https://www.cia.gov/library/publications/the-world-factbook/index.html and https://www.cia.gov/library/publications/cia-maps-publications/index.html

NASA Earth Observatory (public domain): http://earthobservatory.nasa.gov/

Landsat: http://landsat.visibleearth.nasa.gov/

USGS EROS (Earth Resources Observatory and Science (EROS) Center) (public domain): http://eros.usgs.gov/#

Natural Earth (public domain): <http://www.naturalearthdata.com/>

5. Please upload a new copy of Figures 2 to 11 as the detail is not clear. Please follow the link for more information:  <https://journals.plos.org/plosone/s/figures>

6. If the reviewer comments include a recommendation to cite specific previously published works, please review and evaluate these publications to determine whether they are relevant and should be cited. There is no requirement to cite these works unless the editor has indicated otherwise.

**Response:** We thank the Academic Editor and reviewers for their constructive comments. We have substantially revised the manuscript to improve clarity, simplify the presentation of results, and better frame the simulations as a tradeoff-evaluation framework rather than a search for a single optimal scenario. We have also addressed the journal requirements regarding figure quality, map permissions, data availability, and code sharing.

We have prepared the author-generated R code underlying the simulations, analyses, and figures for public release. The code will be made available without restriction upon publication, and the Code Availability Statement has been revised accordingly.

We have revised the Data Availability Statement to include direct links/DOIs for all datasets used in the analysis where available. Derived input files and model outputs necessary to reproduce the analysis will be deposited with the code repository upon publication.

We replaced Figure 1 with a newly generated map using public-domain Natural Earth land polygons and NOAA bathymetry accessed through the marmap R package. The revised caption includes the source information. This replacement figure does not rely on proprietary or copyrighted base-map imagery.

Reviewers' comments:

Reviewer's Responses to Questions

**Comments to the Author**

1. Is the manuscript technically sound, and do the data support the conclusions?

The manuscript must describe a technically sound piece of scientific research with data that supports the conclusions. Experiments must have been conducted rigorously, with appropriate controls, replication, and sample sizes. The conclusions must be drawn appropriately based on the data presented.

Reviewer #1: Partly

Reviewer #2: Partly

2. Has the statistical analysis been performed appropriately and rigorously?

Reviewer #1: N/A

Reviewer #2: No

3. Have the authors made all data underlying the findings in their manuscript fully available?

The [PLOS Data policy](http://www.plosone.org/static/policies.action#sharing) requires authors to make all data underlying the findings described in their manuscript fully available without restriction, with rare exception (please refer to the Data Availability Statement in the manuscript PDF file). The data should be provided as part of the manuscript or its supporting information, or deposited to a public repository. For example, in addition to summary statistics, the data points behind means, medians and variance measures should be available. If there are restrictions on publicly sharing data—e.g. participant privacy or use of data from a third party—those must be specified.

Reviewer #1: Yes

Reviewer #2: Yes

4. Is the manuscript presented in an intelligible fashion and written in standard English?

PLOS ONE does not copyedit accepted manuscripts, so the language in submitted articles must be clear, correct, and unambiguous. Any typographical or grammatical errors should be corrected at revision, so please note any specific errors here.

Reviewer #1: Yes

Reviewer #2: Yes

5. Review Comments to the Author

Please use the space provided to explain your answers to the questions above. You may also include additional comments for the author, including concerns about dual publication, research ethics, or publication ethics. (Please upload your review as an attachment if it exceeds 20,000 characters)

Reviewer #1: This paper addresses important, interesting, and timely questions about fishery management, and this reviewer finds the methods, at a fundamental level, appropriate and well implemented. My serious concerns are about the presentation and interpretation of the results. The authors “tie themselves into knots” by simulating myriads of possibilities and contorting results though scaling, weighting, and intractable/implicit aggregation. Due to all the transformations, I suspect the authors---like anyone reading the paper would---lost track of the correct interpretation of the results. One grave example: Scenario s5 is identified as achieving “the highest overall performance” and “but showed limited improvements in spawning biomass (Figure 6)” (Line 325). As far as this reviewer can ‘track’ the method to this result, it is technically wrong and leads to blatantly wrong/misguided interpretations. IF s5 scores “0.00” for biomass (B) (Table 5 and Figure 6) on a rescaled 0-1 scale, then the proper interpretation of zero is closer to total resource destruction than “limited improvements”!

The authors need to revisit how and what results they generate with a strong focus on simplicity, clarity, and tractability (for the reader). Simulation models provide empirically-guided conceptual results, hence the results presented do not need to cover all possible scenarios but should focus on those limited number of scenarios or permutations that produce interesting insights for fishery management! Much less, would be much more! The interpretation of those result needs to be conditioned by the assumptions/transformations along the way.

**Response:** We thank the reviewer for recognizing the importance of the question and the appropriateness of the underlying modeling framework. We also appreciate the reviewer’s strong concern about the original presentation and interpretation of the results. We agree that the previous version placed too much emphasis on transformed, scaled, and aggregated performance scores, which made the results difficult to interpret and led to overstatement of some scenario-level conclusions.

In response, we have substantially revised the Results and Discussion to emphasize transparent tradeoffs rather than a single overall ranking. Specifically, we have:

1. reorganized the original 16 scenarios into clearly defined scenario families: system-wide effort reduction, recreational effort reduction, recreational effort reduction with hypothetical full retention, fixed spatial closures, and dynamic spatial management;
2. revised the Results to focus on biological, fishery, and economic tradeoffs among scenario families rather than identifying a single “best” scenario;
3. removed the previous interpretation that scenario s5 achieved the “highest overall performance” and no longer describe any scenario as best overall;
4. replaced the previous primary emphasis on the 0–1 rescaled aggregate score with figures showing actual percent changes in biological/fishery metrics and actual economic changes in 2023 U.S. dollars;
5. moved aggregate scoring to an illustrative sensitivity analysis of management weighting assumptions;
6. added clearer figure captions and text explaining that percent changes are shown directly that dead discard reductions have the opposite management interpretation from landings or biomass increases, and that capped color scales are used only for visualization;
7. revised the Discussion to state that scenario rankings depend on management priorities and weighting assumptions, not on inherent properties of the model outputs.

We agree with the reviewer’s specific point regarding scenario s5. In the original manuscript, describing s5 as having “limited improvements in spawning biomass” was incorrect and misleading. In the revised manuscript, we now state that s5 increased recreational landings but produced a negative mean spawning biomass response and reduced spawning biomass for some species, including red snapper.

We have retained the full scenario set because it allows comparison among temporal, spatial, and spatiotemporal input-control mechanisms, but we now present the scenarios as structured management experiments rather than as a large set of competing policy recommendations. We believe this revision better reflects the conceptual role of the simulation model: to reveal tradeoffs among management mechanisms under specified assumptions, not to identify a single optimal policy.

More detailed comments in no particular order:

- Com_net metric represents gross ex-vessel revenue rather than net revenue; that is disingenuous. More materially, Com_net is probably redundant (as Figure 11 also shows), as Recreational Consumer Surplus is (only) comparable with Commercial Producer Surplus. Comparing Producer Surplus to Consumer Surplus is the standard way to evaluate economic tradeoffs between commercial and recreational sectors. Adding another metric adds nothing (except confusion).

**Response:** We revised the Methods, Results, figure labels, and table captions to rename Com_net as commercial gross ex-vessel revenue, clarify that it is not a net metric. We retained commercial gross ex-vessel revenue as a supplemental descriptive metric of commercial dockside value while focusing the economic tradeoff interpretation on producer surplus and consumer surplus.

- If I understand it correctly, discards, valued at $10, can become catch, valued at $100, in some full retention scenarios (e.g., two very red boxes in Fig 8). This is conceptually flawed as discarding happens for many reasons—such as fish simply being too small or low quality to keep—not just because of regulatory limits. It’s a delicate, complicated situation---and this research might shed some light on that---or not if it's not a central result, in which case Rec_D_cs could probably be dropped from the paper entirely (again, to simplify and focus on central results without distraction, i.e., two econ metrics would probably do).

**Response:** We agree with the reviewer that discards are complicated and that not all discarded fish could realistically be converted into retained catch. Fish may be discarded because they are undersized, damaged, low quality, undesirable to anglers, or subject to other regulatory or behavioral constraints. We have revised the manuscript to clarify this point and to avoid implying that all discards would become retained catch under the full-retention scenarios.

Our intent was to evaluate changes in the economic value associated with regulatory discards under a simplified retention framework. In the model, full retention does not completely eliminate discards. Rather, it is implemented by setting the asymptote of the recreational retention ogive to one while retaining the length-dependent logistic retention curve. Thus, small fish can still be discarded because retention remains length-dependent, whereas large fish are no longer subject to an asymptotic cap on retention.

- Rescaling of metrics always involves a loss of interpretability and should be used sparingly and only when necessary. Rescaling to 0-1 loses cardinality and truncation, etc. Rescaling and then adding up is even more delicate. Related, this reviewer highly dislikes the radar plots (Figures 4 and 6), as they obscure results rather than showing any. If ONE radar plot were kept, e.g., figure 6, a status quo/baseline line/polygon MUST be added to the plot to show the direction of changes!!! (you would have seen what a complete Biomass disaster s5 is)

**Response:** We agree with the reviewer. In response, we removed the radar plots from the main manuscript and replaced them with heatmaps and dot plots that show more directly interpretable quantities. Biological and fishery outcomes are now presented as percent changes relative to status quo, and economic outcomes are presented as changes in 2023 U.S. dollars.

We no longer use the rescaled aggregate score as a primary basis for interpreting scenario performance. Instead, the Results are organized around tradeoffs among scenario families, species, sectors, and metrics. Any rescaled scoring is now limited to an illustrative sensitivity analysis that evaluates how scenario scores change under alternative management weighting assumptions.

We also agree with the reviewer’s specific point about scenario s5. The revised Results now state that s5 increased recreational landings but produced a negative mean spawning biomass response and reduced spawning biomass for some species. We no longer describe s5 as achieving the highest overall performance or as showing “limited improvements” in biomass.

- Rescaling $ values (fig 8) is rescaling a ‘scale’, since $ is already a monetization of other units/dimensions. It shouldn’t be necessary, and results would be much easier to interpret, e.g., top left number in Fig 8 is “-0.1”---is this minus $100,000? Minus $10? And would “-0.2” be double the loss???

**Response:** We removed the rescaled 0–1 economic figure and replaced it with figures and tables that report economic results in dollar units.

- Regarding Table 6 and associated (not well documented) analysis: Computing a single aggregated Score across all metrics relies on the very strong assumption that all five performance metrics are valued equally at 20%. Fishery management laws clearly state biomass can’t be fully compromised! The authors must carefully evaluate how to proceed with aggregation and then clearly state their assumptions (and why) in the text. I have no faith that the currently calculated Rank provides any value for fishery management (because 'equal 20%' IS a massive assumption (about policy goals), even if not documented)!

**Response:** We agree with the reviewer. In response, we removed the original Table 6 and no longer use a single aggregate rank as a primary result or as the basis for interpreting scenario performance. The revised Results instead present the biological, fishery, and economic metrics separately and emphasize tradeoffs among scenario families. Spawning biomass, landings, and dead discards are now shown directly as percent changes relative to status quo, and economic metrics are shown in dollar units. We also revised the Discussion to state explicitly that selecting a preferred scenario would require explicit management objectives, legal constraints, and stakeholder weighting of competing outcomes.

Because weighting assumptions can still be useful for illustrating how management priorities affect scenario interpretation, we retained an aggregate score only as a clearly labeled illustrative sensitivity analysis. In that analysis, we compare multiple weighting schemes, including conservation-heavy, harvest-heavy, discard-reduction-heavy, and equal weighting. We explicitly state that these scores are not management recommendations and should not be interpreted as identifying a preferred policy. Rather, their purpose is to demonstrate that scenario rankings are sensitive to policy weights and that no single ranking is inherent to the model outputs.

- There are too many scenarios, especially in light of the (effort) sensitivity analysis later. FOCUS. Are north/south regions or inshore/offshore really necessary for the central results of this paper? Alternatively, put the huge number of permutations into an appendix while keeping only the most interesting cases in the main text, tables, and figures.

**Response:** We agree that the original presentation made the number of scenarios difficult to follow, particularly when combined with the effort-reduction sensitivity analysis. We have revised the manuscript to improve focus and to make clear why the full scenario set was included.

We retained the original 16 scenarios because they were designed to represent distinct management mechanisms rather than arbitrary permutations. The north/middle/south and nearshore/offshore scenarios are relevant because the model is spatially explicit and species distributions, fishing effort, and discard mortality vary across both latitude and depth. Thus, the regional and depth-based scenarios help evaluate whether spatial input controls can produce different tradeoffs from broad temporal effort reductions. However, we agree that treating all scenarios as equally central obscured the main message.

In response, we reorganized the scenarios into clearly defined scenario families: system-wide effort reduction, recreational effort reduction, recreational effort reduction with hypothetical full retention, fixed spatial closures, and dynamic spatial management. We also revised the Results so that the main text focuses on the most important contrasts among scenario families rather than describing every scenario individually.

We also clarified the relationship between the 16 management scenarios and the effort sensitivity analysis. The 16 scenarios evaluate qualitatively different management mechanisms, including spatial and dynamic spatial controls, whereas the sensitivity analysis provides a higher-resolution evaluation of effort-reduction intensity for the temporal scenarios. Thus, the sensitivity analysis does not replace the spatial scenarios; rather, it complements them by showing how outcomes vary with the magnitude of effort reduction.

- Figure 3: Use reverse color-coding for discards here too?

**Response:** We revised the caption for Figure 3 to state: “For spawning biomass and landings, positive values indicate increases. For dead discards, negative values indicate reductions in discard mortality.”

- Methodology should be in the text, not the table headings, including any rescaling, etc. Once you write it out in the text, you’ll realize how much of it does not enhance understanding…

**Response:** We agree with the reviewer and made the changes accordingly.

- I think there is an internal inconsistency in Fig 5 regarding Biomass compared to Fig 3. Biomass should improve in most scenarios, no (but in Fig 5 all cells are blue)? More generally, both tables look at by species, by scenario, by metric (for a lot of permutations) but are ‘transposed’ in a different manner. Just makes it unnecessarily hard for the reader to follow. Consistency!

**Response:** We agree with the reviewer. The previous figures were unnecessarily difficult to compare because similar information was presented in different orientations and with different transformations. This made it hard to track scenario-, species-, and metric-specific responses across figures and likely contributed to the apparent inconsistency noted by the reviewer. In response, we removed the previous rescaled performance figure and replaced it with figures that show direct, interpretable quantities. Biological and fishery outcomes are now shown consistently as percent changes relative to status quo.

- Steepness of 0.99? Doesn’t that mean recruitment is basically independent of biomass? Why would managing catch and discards matter, real world, but also mathematically?

**Response:** Yes, a steepness value of 0.99 implies recruitment is nearly independent of spawning biomass over much of the modeled range. We used these values to remain consistent with the species-specific stock assessments.

- Replace the ambiguous term "prevailing" with "base-level" or "status quo."

**Response:** We have made the changes as suggested by the reviewer.

- Line 49: From an economic perspective, fishery management falls into two categories, “market based” and “command and control.” Output or input controls are both in the latter group. Market-based or ‘rights-based’ fishery management are ITQs, IFQs, or catch shares.

**Response:** We agree with the reviewer that from an economic perspective, fishery management falls into those two categories.

- Line 72: “closures” instead of “openings”?

**Response:** We have made the changes as suggested by the reviewer.

- Line 17: typo ‘egive’?

**Response:** We have made the changes as suggested by the reviewer.

- Line 226: The final “TWO” scenario”S”?

**Response:** We have made the changes as suggested by the reviewer.

- Line 244/245: I think the base case is ‘0’ and there are 16 proposed management scenarios?

**Response:** Yes, the reviewer is correct.

- Line 255: Is there any conversion between commercial lbs of fish and recreational numbers of fish? Is that explained in the text, and I overlooked it?

**Response:** No, we are using numbers of fish for calculating recreational economic values.

- Line 260: I think the cite should be: Liese, C. 2023. Economics of the U.S. South Atlantic Snapper-Grouper Fishery - 2018. NOAA Technical Memorandum NMFS-SEFSC-774.

**Response:** We have added this citation.

- Table 5: Rec_L_cs and Rec_D-cs should be rounded to dollars, the precision implied by reporting cents does not hold for the underlying recreational research cited.

**Response:** We have made the changes as suggested by the reviewer.

Reviewer #2: This study used a simulation model to examine the effectiveness of alternative temporal and spatial management strategies on the multispecies snapper-grouper fishery in the southeast United States Atlantic. It evaluated performance metrics including spawning biomass, commercial and recreational landings, commercial and recreational discards, and economic indicators for six species within the complex. The results show that different management strategies work better for different species, highlighting the challenges inherent to managing a multispecies fishery. The paper is written clearly and concisely.

The paper includes multiple unrealistic assumptions that the authors readily acknowledge, including (i) a focus on equilibrium outcomes in deterministic simulations without explicit treatment of uncertainty and (ii) an assumption that there would be no reallocation of fishing effort under almost all of the alternative temporal and spatial management scenarios. Both assumptions are addressed extensively in the fisheries literature, and it is well known that violation of these assumptions commonly leads to qualitatively different outcomes and inferences. The use of point-estimate parameters (Table 1) is especially concerning. While I understand that this study is being published posthumously for one of the co-lead authors and it may not be possible to augment the analytical framework, these issues are major concerns.

**Response:** We thank the reviewer for this careful assessment and agree that deterministic equilibrium simulations, point-estimate parameters, and simplified effort redistribution are important limitations. We have revised the manuscript to make these limitations more explicit and to narrow the interpretation of the results accordingly.

Our intent was not to conduct a full management strategy evaluation or to forecast short-term outcomes following implementation. Rather, the model was used as a comparative operating-model framework to evaluate the direction and relative magnitude of long-term tradeoffs among management mechanisms under a common set of assumptions. We now state this more clearly in the Methods and Discussion. Specifically, we clarify that the simulations estimate deterministic equilibrium outcomes and should be interpreted as expected long-term comparisons, not as predictions of transient responses or policy performance under uncertainty.

Regarding effort redistribution, we have clarified that behavioral redistribution was represented only in the offshore-closure scenarios, where offshore effort was shifted nearshore. For other spatial scenarios, effort outside closed areas was maintained at baseline, and additional redistribution was not modeled. We agree that this simplifying assumption could change projected outcomes if fishers respond by shifting effort across space, time, target species, or sectors. We now explicitly state that the spatial scenarios should be interpreted as structured comparisons of access restrictions, not as forecasts of fisher behavior after implementation.

I note that all scenarios lead to extremely poor performance for recreational and commercial landings (Figs 4-5). This result is potentially a consequence of assuming no reallocation of fishing effort. More importantly, it is therefore unclear why fisheries managers and the fishing industries would consider these scenarios. Is it possible to identify and explore a greater range of scenarios, including those where recreational and commercial landings might be similar to or better than the status quo? If not, why not, and can the authors provide some justification for where the legal mandate(s) and/or political will might emerge to pursue the scenarios they chose?

**Response:** The original figures could give the impression that nearly all scenarios performed poorly for landings. This was partly a consequence of the previous figures based on rescaled performance metrics, which obscured the direction and magnitude of the actual changes. We have removed those figures from the revised manuscript and replaced them with figures showing direct percent changes in biological and fishery metrics relative to status quo.

In the revised results, it is clearer that several scenarios increased commercial and/or recreational landings. For example, the full-retention scenarios increased recreational landings, while several recreational-only effort reduction and spatial closure scenarios increased commercial landings because commercial effort was maintained while recreational fishing mortality was reduced. The rotating regional opening scenario also increased commercial landings, although this came with increased commercial dead discards. We now present these outcomes as tradeoffs rather than as universally poor landings performance.

The paper did not explicitly consider the potential for interactions (eg, competition) between the 6 species under consideration, or the effects of habitat (eg, structure), on species dynamics or the outcomes of the alternative management strategies. This omission strikes me as a missed opportunity, and could be suggested as a future direction.

**Response:** We agree with the reviewer. The current operating model represents the six species simultaneously and allows them to differ in life history, spatial distribution, selectivity, retention, discard mortality, and fleet interactions, but it does not explicitly model ecological interactions among species, such as competition, predation, or shared habitat-mediated density dependence. Habitat effects are represented only indirectly through the spatial distribution of abundance and fishing effort across the six model areas, rather than through explicit habitat covariates or reef-structure dynamics. We have revised the Discussion to acknowledge this limitation and to identify it as an important future direction.

The figures require a lot of the reader, with so many scenarios and response variables represented by cryptic acronyms and the application of difficult color palettes to represent the scenarios in the radar plots. I encourage the authors to heavily revise the figures so that the acronyms are spelled out in the figures and the distinctions among scenarios in the radar plots are made more obvious.

**Response:** We agree with the reviewer. The original figure set required too much interpretation from the reader, particularly because several response variables were shown as acronyms, scenario distinctions were difficult to follow, and the radar plots were visually complex.

In response, we substantially revised the figure set. We removed the radar plots from the main manuscript and replaced them with heatmaps and dot plots that show more directly interpretable quantities. Biological and fishery outcomes are now shown as percent changes relative to status quo, and economic outcomes are shown as changes in 2023 U.S. dollars. We also replaced cryptic metric labels with clearer labels, such as “Spawning biomass,” “Rec landings,” “Com landings,” “Rec dead discards,” and “Com dead discards.”
