# Supplementary material for: Navigating complexity: Evaluating management measures for multispecies snapper-grouper fisheries in the Southeast U.S. Atlantic
Source: PLoS One. 2026 Aug 14;21(8):e0355048. doi: 10.1371/journal.pone.0355048 (PMC13475873; doi:10.1371/journal.pone.0355048)
Supplement: Peer review history

# Peer review: Decision Letter 0

**DOI:** 10.1371/journal.pone.0355048.r001

**Authors:** Abdul Azeez Pokkathappada

5 Apr 2026

Dear Dr. Cao,

Thank you for submitting your manuscript to PLOS ONE. After careful consideration, we feel that it has merit but does not fully meet PLOS ONE’s publication criteria as it currently stands. Therefore, we invite you to submit a revised version of the manuscript that addresses the points raised during the review process.

Please submit your revised manuscript by May 20 2026 11:59PM. If you will need more time than this to complete your revisions, please reply to this message or contact the journal office at plosone@plos.org. When you're ready to submit your revision, log on to https://www.editorialmanager.com/pone/ and select the 'Submissions Needing Revision' folder to locate your manuscript file.

- A letter that responds to each point raised by the academic editor and reviewer(s). You should upload this letter as a separate file labeled 'Response to Reviewers'.
- A marked-up copy of your manuscript that highlights changes made to the original version. You should upload this as a separate file labeled 'Revised Manuscript with Track Changes'.
- An unmarked version of your revised paper without tracked changes. You should upload this as a separate file labeled 'Manuscript'.

If you would like to make changes to your financial disclosure, please include your updated statement in your cover letter. Guidelines for resubmitting your figure files are available below the reviewer comments at the end of this letter.

If applicable, we recommend that you deposit your laboratory protocols in protocols.io to enhance the reproducibility of your results. Protocols.io assigns your protocol its own identifier (DOI) so that it can be cited independently in the future. For instructions see: https://journals.plos.org/plosone/s/submission-guidelines#loc-laboratory-protocols. Additionally, PLOS ONE offers an option for publishing peer-reviewed Lab Protocol articles, which describe protocols hosted on protocols.io. Read more information on sharing protocols at https://plos.org/protocols?utm_medium=editorial-email&utm_source=authorletters&utm_campaign=protocols.

As the corresponding author, your ORCID iD is verified in the submission system and will appear in the published article. PLOS supports the use of ORCID, and we encourage all coauthors to register for an ORCID iD and use it as well. Please encourage your coauthors to verify their ORCID iD within the submission system before final acceptance, as unverified ORCID iDs will not appear in the published article. *Only*  the individual author can complete the verification step; PLOS staff *cannot*  verify ORCID iDs on behalf of authors.

We look forward to receiving your revised manuscript.

Kind regards,

Abdul Azeez Pokkathappada, Ph.D.

Academic Editor

PLOS One

**Journal Requirements:**

When submitting your revision, we need you to address these additional requirements.

1. Please ensure that your manuscript meets PLOS ONE's style requirements, including those for file naming. The PLOS ONE style templates can be found at

https://journals.plos.org/plosone/s/file?id=wjVg/PLOSOne_formatting_sample_main_body.pdf and

https://journals.plos.org/plosone/s/file?id=ba62/PLOSOne_formatting_sample_title_authors_affiliations.pdf

2. Please note that PLOS One has specific guidelines on code sharing for submissions in which author-generated code underpins the findings in the manuscript. In these cases, we expect all author-generated code to be made available without restrictions upon publication of the work. Please review our guidelines at https://journals.plos.org/plosone/s/materials-and-software-sharing#loc-sharing-code and ensure that your code is shared in a way that follows best practice and facilitates reproducibility and reuse.

3. Please note that your Data Availability Statement is currently missing the DOI/accession number of each dataset OR a direct link to access each database. If your manuscript is accepted for publication, you will be asked to provide these details on a very short timeline. We therefore suggest that you provide this information now, though we will not hold up the peer review process if you are unable.

4. We note that Figure 1 in your submission contain map images which may be copyrighted. All PLOS content is published under the Creative Commons Attribution License (CC BY 4.0), which means that the manuscript, images, and Supporting Information files will be freely available online, and any third party is permitted to access, download, copy, distribute, and use these materials in any way, even commercially, with proper attribution. For these reasons, we cannot publish previously copyrighted maps or satellite images created using proprietary data, such as Google software (Google Maps, Street View, and Earth). For more information, see our copyright guidelines: http://journals.plos.org/plosone/s/licenses-and-copyright.

We require you to either (1) present written permission from the copyright holder to publish these figures specifically under the CC BY 4.0 license, or (2) remove the figures from your submission:

1. You may seek permission from the original copyright holder of Figure 1 to publish the content specifically under the CC BY 4.0 license.

We recommend that you contact the original copyright holder with the Content Permission Form (http://journals.plos.org/plosone/s/file?id=7c09/content-permission-form.pdf) and the following text:

“I request permission for the open-access journal PLOS ONE to publish XXX under the Creative Commons Attribution License (CCAL) CC BY 4.0 (http://creativecommons.org/licenses/by/4.0/). Please be aware that this license allows unrestricted use and distribution, even commercially, by third parties. Please reply and provide explicit written permission to publish XXX under a CC BY license and complete the attached form.”

Please upload the completed Content Permission Form or other proof of granted permissions as an "Other" file with your submission.

In the figure caption of the copyrighted figure, please include the following text: “Reprinted from [ref] under a CC BY license, with permission from [name of publisher], original copyright [original copyright year].”

2. If you are unable to obtain permission from the original copyright holder to publish these figures under the CC BY 4.0 license or if the copyright holder’s requirements are incompatible with the CC BY 4.0 license, please either i) remove the figure or ii) supply a replacement figure that complies with the CC BY 4.0 license. Please check copyright information on all replacement figures and update the figure caption with source information. If applicable, please specify in the figure caption text when a figure is similar but not identical to the original image and is therefore for illustrative purposes only.

The following resources for replacing copyrighted map figures may be helpful:

USGS National Map Viewer (public domain): http://viewer.nationalmap.gov/viewer/

The Gateway to Astronaut Photography of Earth (public domain): http://eol.jsc.nasa.gov/sseop/clickmap/

Maps at the CIA (public domain): https://www.cia.gov/library/publications/the-world-factbook/index.html and https://www.cia.gov/library/publications/cia-maps-publications/index.html

NASA Earth Observatory (public domain): http://earthobservatory.nasa.gov/

Landsat: http://landsat.visibleearth.nasa.gov/

USGS EROS (Earth Resources Observatory and Science (EROS) Center) (public domain): http://eros.usgs.gov/#

Natural Earth (public domain): http://www.naturalearthdata.com/

5. Please upload a new copy of Figures 2 to 11 as the detail is not clear. Please follow the link for more information:  https://journals.plos.org/plosone/s/figures

6. If the reviewer comments include a recommendation to cite specific previously published works, please review and evaluate these publications to determine whether they are relevant and should be cited. There is no requirement to cite these works unless the editor has indicated otherwise.

[Note: HTML markup is below. Please do not edit.]

Reviewers' comments:

Reviewer's Responses to Questions

**Comments to the Author**

1. Is the manuscript technically sound, and do the data support the conclusions?

Reviewer #1: Partly

Reviewer #2: Partly

**********

2. Has the statistical analysis been performed appropriately and rigorously?

Reviewer #1: N/A

Reviewer #2: No

**********

3. Have the authors made all data underlying the findings in their manuscript fully available?

The PLOS Data policy

Reviewer #1: Yes

Reviewer #2: Yes

**********

4. Is the manuscript presented in an intelligible fashion and written in standard English?

Reviewer #1: Yes

Reviewer #2: Yes

**********

Reviewer #1: This paper addresses important, interesting, and timely questions about fishery management, and this reviewer finds the methods, at a fundamental level, appropriate and well implemented. My serious concerns are about the presentation and interpretation of the results. The authors “tie themselves into knots” by simulating myriads of possibilities and contorting results though scaling, weighting, and intractable/implicit aggregation. Due to all the transformations, I suspect the authors---like anyone reading the paper would---lost track of the correct interpretation of the results. One grave example: Scenario s5 is identified as achieving “the highest overall performance” and “but showed limited improvements in spawning biomass (Figure 6)” (Line 325). As far as this reviewer can ‘track’ the method to this result, it is technically wrong and leads to blatantly wrong/misguided interpretations. IF s5 scores “0.00” for biomass (B) (Table 5 and Figure 6) on a rescaled 0-1 scale, then the proper interpretation of zero is closer to total resource destruction than “limited improvements”!

The authors need to revisit how and what results they generate with a strong focus on simplicity, clarity, and tractability (for the reader). Simulation models provide empirically-guided conceptual results, hence the results presented do not need to cover all possible scenarios but should focus on those limited number of scenarios or permutations that produce interesting insights for fishery management! Much less, would be much more! The interpretation of those result needs to be conditioned by the assumptions/transformations along the way.

More detailed comments in no particular order:

- Com_net metric represents gross ex-vessel revenue rather than net revenue; that is disingenuous. More materially, Com_net is probably redundant (as Figure 11 also shows), as Recreational Consumer Surplus is (only) comparable with Commercial Producer Surplus. Comparing Producer Surplus to Consumer Surplus is the standard way to evaluate economic tradeoffs between commercial and recreational sectors. Adding another metric adds nothing (except confusion).

- If I understand it correctly, discards, valued at $10, can become catch, valued at $100, in some full retention scenarios (e.g., two very red boxes in Fig 8). This is conceptually flawed as discarding happens for many reasons—such as fish simply being too small or low quality to keep—not just because of regulatory limits. It’s a delicate, complicated situation---and this research might shed some light on that---or not if it's not a central result, in which case Rec_D_cs could probably be dropped from the paper entirely (again, to simplify and focus on central results without distraction, i.e., two econ metrics would probably do).

- Rescaling of metrics always involves a loss of interpretability and should be used sparingly and only when necessary. Rescaling to 0-1 loses cardinality and truncation, etc. Rescaling and then adding up is even more delicate. Related, this reviewer highly dislikes the radar plots (Figures 4 and 6), as they obscure results rather than showing any. If ONE radar plot were kept, e.g., figure 6, a status quo/baseline line/polygon MUST be added to the plot to show the direction of changes!!! (you would have seen what a complete Biomass disaster s5 is)

- Rescaling $ values (fig 8) is rescaling a ‘scale’, since $ is already a monetization of other units/dimensions. It shouldn’t be necessary, and results would be much easier to interpret, e.g., top left number in Fig 8 is “-0.1”---is this minus $100,000? Minus $10? And would “-0.2” be double the loss???

- Regarding Table 6 and associated (not well documented) analysis: Computing a single aggregated Score across all metrics relies on the very strong assumption that all five performance metrics are valued equally at 20%. Fishery management laws clearly state biomass can’t be fully compromised! The authors must carefully evaluate how to proceed with aggregation and then clearly state their assumptions (and why) in the text. I have no faith that the currently calculated Rank provides any value for fishery management (because 'equal 20%' IS a massive assumption (about policy goals), even if not documented)!

- There are too many scenarios, especially in light of the (effort) sensitivity analysis later. FOCUS. Are north/south regions or inshore/offshore really necessary for the central results of this paper? Alternatively, put the huge number of permutations into an appendix while keeping only the most interesting cases in the main text, tables, and figures.

- Figure 3: Use reverse color-coding for discards here too?

- Methodology should be in the text, not the table headings, including any rescaling, etc. Once you write it out in the text, you’ll realize how much of it does not enhance understanding…

- I think there is an internal inconsistency in Fig 5 regarding Biomass compared to Fig 3. Biomass should improve in most scenarios, no (but in Fig 5 all cells are blue)? More generally, both tables look at by species, by scenario, by metric (for a lot of permutations) but are ‘transposed’ in a different manner. Just makes it unnecessarily hard for the reader to follow. Consistency!

- Steepness of 0.99? Doesn’t that mean recruitment is basically independent of biomass? Why would managing catch and discards matter, real world, but also mathematically?

- Replace the ambiguous term "prevailing" with "base-level" or "status quo."

- Line 49: From an economic perspective, fishery management falls into two categories, “market based” and “command and control.” Output or input controls are both in the latter group. Market-based or ‘rights-based’ fishery management are ITQs, IFQs, or catch shares.

- Line 72: “closures” instead of “openings”?

- Line 17: typo ‘egive’?

- Line 226: The final “TWO” scenario”S”?

- Line 244/245: I think the base case is ‘0’ and there are 16 proposed management scenarios?

- Line 255: Is there any conversion between commercial lbs of fish and recreational numbers of fish? Is that explained in the text, and I overlooked it?

- Line 260: I think the cite should be: Liese, C. 2023. Economics of the U.S. South Atlantic Snapper-Grouper Fishery - 2018. NOAA Technical Memorandum NMFS-SEFSC-774.

- Table 5: Rec_L_cs and Rec_D-cs should be rounded to dollars, the precision implied by reporting cents does not hold for the underlying recreational research cited.

Reviewer #2: This study used a simulation model to examine the effectiveness of alternative temporal and spatial management strategies on the multispecies snapper-grouper fishery in the southeast United States Atlantic. It evaluated performance metrics including spawning biomass, commercial and recreational landings, commercial and recreational discards, and economic indicators for six species within the complex. The results show that different management strategies work better for different species, highlighting the challenges inherent to managing a multispecies fishery. The paper is written clearly and concisely.

The paper includes multiple unrealistic assumptions that the authors readily acknowledge, including (i) a focus on equilibrium outcomes in deterministic simulations without explicit treatment of uncertainty and (ii) an assumption that there would be no reallocation of fishing effort under almost all of the alternative temporal and spatial management scenarios. Both assumptions are addressed extensively in the fisheries literature, and it is well known that violation of these assumptions commonly leads to qualitatively different outcomes and inferences. The use of point-estimate parameters (Table 1) is especially concerning. While I understand that this study is being published posthumously for one of the co-lead authors and it may not be possible to augment the analytical framework, these issues are major concerns.

I note that all scenarios lead to extremely poor performance for recreational and commercial landings (Figs 4-5). This result is potentially a consequence of assuming no reallocation of fishing effort. More importantly, it is therefore unclear why fisheries managers and the fishing industries would consider these scenarios. Is it possible to identify and explore a greater range of scenarios, including those where recreational and commercial landings might be similar to or better than the status quo? If not, why not, and can the authors provide some justification for where the legal mandate(s) and/or political will might emerge to pursue the scenarios they chose?

The paper did not explicitly consider the potential for interactions (eg, competition) between the 6 species under consideration, or the effects of habitat (eg, structure), on species dynamics or the outcomes of the alternative management strategies. This omission strikes me as a missed opportunity, and could be suggested as a future direction.

The figures require a lot of the reader, with so many scenarios and response variables represented by cryptic acronyms and the application of difficult color palettes to represent the scenarios in the radar plots. I encourage the authors to heavily revise the figures so that the acronyms are spelled out in the figures and the distinctions among scenarios in the radar plots are made more obvious.

**********

what does this mean?). If published, this will include your full peer review and any attached files.

If you choose “no”, your identity will remain anonymous but your review may still be made public.

**Do you want your identity to be public for this peer review?**  For information about this choice, including consent withdrawal, please see our Privacy Policy

Reviewer #1: No

Reviewer #2: No

**********

[NOTE: If reviewer comments were submitted as an attachment file, they will be attached to this email and accessible via the submission site. Please log into your account, locate the manuscript record, and check for the action link "View Attachments". If this link does not appear, there are no attachment files.]

To ensure your figures meet our technical requirements, please review our figure guidelines: https://journals.plos.org/plosone/s/figures

You may also use PLOS’s free figure tool, NAAS, to help you prepare publication quality figures: https://journals.plos.org/plosone/s/figures#loc-tools-for-figure-preparation.

NAAS will assess whether your figures meet our technical requirements by comparing each figure against our figure specifications.

# Author comment: Author response to Decision Letter 1

**DOI:** 10.1371/journal.pone.0355048.r002

1 Jun 2026

PONE-D-26-09475

Navigating Complexity: Evaluating Management Measures for Multispecies Snapper-Grouper Fisheries in the Southeast U.S. Atlantic

PLOS One

Dear Dr. Cao,

Thank you for submitting your manuscript to PLOS ONE. After careful consideration, we feel that it has merit but does not fully meet PLOS ONE’s publication criteria as it currently stands. Therefore, we invite you to submit a revised version of the manuscript that addresses the points raised during the review process.

Please submit your revised manuscript by May 20 2026 11:59PM. If you will need more time than this to complete your revisions, please reply to this message or contact the journal office at plosone@plos.org. When you're ready to submit your revision, log on to https://www.editorialmanager.com/pone/ and select the 'Submissions Needing Revision' folder to locate your manuscript file.

Please include the following items when submitting your revised manuscript:

• A letter that responds to each point raised by the academic editor and reviewer(s). You should upload this letter as a separate file labeled 'Response to Reviewers'.

• A marked-up copy of your manuscript that highlights changes made to the original version. You should upload this as a separate file labeled 'Revised Manuscript with Track Changes'.

• An unmarked version of your revised paper without tracked changes. You should upload this as a separate file labeled 'Manuscript'.

If you would like to make changes to your financial disclosure, please include your updated statement in your cover letter. Guidelines for resubmitting your figure files are available below the reviewer comments at the end of this letter.

If applicable, we recommend that you deposit your laboratory protocols in protocols.io to enhance the reproducibility of your results. Protocols.io assigns your protocol its own identifier (DOI) so that it can be cited independently in the future. For instructions see: https://journals.plos.org/plosone/s/submission-guidelines#loc-laboratory-protocols. Additionally, PLOS ONE offers an option for publishing peer-reviewed Lab Protocol articles, which describe protocols hosted on protocols.io. Read more information on sharing protocols at https://plos.org/protocols?utm_medium=editorial-email&utm_source=authorletters&utm_campaign=protocols.

As the corresponding author, your ORCID iD is verified in the submission system and will appear in the published article. PLOS supports the use of ORCID, and we encourage all coauthors to register for an ORCID iD and use it as well. Please encourage your coauthors to verify their ORCID iD within the submission system before final acceptance, as unverified ORCID iDs will not appear in the published article. Only the individual author can complete the verification step; PLOS staff cannot verify ORCID iDs on behalf of authors.

We look forward to receiving your revised manuscript.

Kind regards,

Abdul Azeez Pokkathappada, Ph.D.

Academic Editor

PLOS One

Journal Requirements:

When submitting your revision, we need you to address these additional requirements.

1. Please ensure that your manuscript meets PLOS ONE's style requirements, including those for file naming. The PLOS ONE style templates can be found at

https://journals.plos.org/plosone/s/file?id=wjVg/PLOSOne_formatting_sample_main_body.pdfand

https://journals.plos.org/plosone/s/file?id=ba62/PLOSOne_formatting_sample_title_authors_affiliations.pdf

2. Please note that PLOS One has specific guidelines on code sharing for submissions in which author-generated code underpins the findings in the manuscript. In these cases, we expect all author-generated code to be made available without restrictions upon publication of the work. Please review our guidelines at https://journals.plos.org/plosone/s/materials-and-software-sharing#loc-sharing-code and ensure that your code is shared in a way that follows best practice and facilitates reproducibility and reuse.

3. Please note that your Data Availability Statement is currently missing the DOI/accession number of each dataset OR a direct link to access each database. If your manuscript is accepted for publication, you will be asked to provide these details on a very short timeline. We therefore suggest that you provide this information now, though we will not hold up the peer review process if you are unable.

4. We note that Figure 1 in your submission contain map images which may be copyrighted. All PLOS content is published under the Creative Commons Attribution License (CC BY 4.0), which means that the manuscript, images, and Supporting Information files will be freely available online, and any third party is permitted to access, download, copy, distribute, and use these materials in any way, even commercially, with proper attribution. For these reasons, we cannot publish previously copyrighted maps or satellite images created using proprietary data, such as Google software (Google Maps, Street View, and Earth). For more information, see our copyright guidelines: http://journals.plos.org/plosone/s/licenses-and-copyright.

We require you to either (1) present written permission from the copyright holder to publish these figures specifically under the CC BY 4.0 license, or (2) remove the figures from your submission:

1. You may seek permission from the original copyright holder of Figure 1 to publish the content specifically under the CC BY 4.0 license.

We recommend that you contact the original copyright holder with the Content Permission Form (http://journals.plos.org/plosone/s/file?id=7c09/content-permission-form.pdf) and the following text:

“I request permission for the open-access journal PLOS ONE to publish XXX under the Creative Commons Attribution License (CCAL) CC BY 4.0 (http://creativecommons.org/licenses/by/4.0/). Please be aware that this license allows unrestricted use and distribution, even commercially, by third parties. Please reply and provide explicit written permission to publish XXX under a CC BY license and complete the attached form.”

Please upload the completed Content Permission Form or other proof of granted permissions as an "Other" file with your submission.

In the figure caption of the copyrighted figure, please include the following text: “Reprinted from [ref] under a CC BY license, with permission from [name of publisher], original copyright [original copyright year].”

2. If you are unable to obtain permission from the original copyright holder to publish these figures under the CC BY 4.0 license or if the copyright holder’s requirements are incompatible with the CC BY 4.0 license, please either i) remove the figure or ii) supply a replacement figure that complies with the CC BY 4.0 license. Please check copyright information on all replacement figures and update the figure caption with source information. If applicable, please specify in the figure caption text when a figure is similar but not identical to the original image and is therefore for illustrative purposes only.

The following resources for replacing copyrighted map figures may be helpful:

USGS National Map Viewer (public domain): http://viewer.nationalmap.gov/viewer/

The Gateway to Astronaut Photography of Earth (public domain): http://eol.jsc.nasa.gov/sseop/clickmap/

Maps at the CIA (public domain): https://www.cia.gov/library/publications/the-world-factbook/index.html and https://www.cia.gov/library/publications/cia-maps-publications/index.html

NASA Earth Observatory (public domain): http://earthobservatory.nasa.gov/

Landsat: http://landsat.visibleearth.nasa.gov/

USGS EROS (Earth Resources Observatory and Science (EROS) Center) (public domain): http://eros.usgs.gov/#

Natural Earth (public domain): http://www.naturalearthdata.com/

5. Please upload a new copy of Figures 2 to 11 as the detail is not clear. Please follow the link for more information: https://journals.plos.org/plosone/s/figures

6. If the reviewer comments include a recommendation to cite specific previously published works, please review and evaluate these publications to determine whether they are relevant and should be cited. There is no requirement to cite these works unless the editor has indicated otherwise.

Response: We thank the Academic Editor and reviewers for their constructive comments. We have substantially revised the manuscript to improve clarity, simplify the presentation of results, and better frame the simulations as a tradeoff-evaluation framework rather than a search for a single optimal scenario. We have also addressed the journal requirements regarding figure quality, map permissions, data availability, and code sharing.

We have prepared the author-generated R code underlying the simulations, analyses, and figures for public release. The code will be made available without restriction upon publication, and the Code Availability Statement has been revised accordingly.

We have revised the Data Availability Statement to include direct links/DOIs for all datasets used in the analysis where available. Derived input files and model outputs necessary to reproduce the analysis will be deposited with the code repository upon publication.

We replaced Figure 1 with a newly generated map using public-domain Natural Earth land polygons and NOAA bathymetry accessed through the marmap R package. The revised caption includes the source information. This replacement figure does not rely on proprietary or copyrighted base-map imagery.

Reviewers' comments:

Reviewer's Responses to Questions

Comments to the Author

1. Is the manuscript technically sound, and do the data support the conclusions?

The manuscript must describe a technically sound piece of scientific research with data that supports the conclusions. Experiments must have been conducted rigorously, with appropriate controls, replication, and sample sizes. The conclusions must be drawn appropriately based on the data presented.

Reviewer #1: Partly

Reviewer #2: Partly

2. Has the statistical analysis been performed appropriately and rigorously?

Reviewer #1: N/A

Reviewer #2: No

3. Have the authors made all data underlying the findings in their manuscript fully available?

The PLOS Data policy requires authors to make all data underlying the findings described in their manuscript fully available without restriction, with rare exception (please refer to the Data Availability Statement in the manuscript PDF file). The data should be provided as part of the manuscript or its supporting information, or deposited to a public repository. For example, in addition to summary statistics, the data points behind means, medians and variance measures should be available. If there are restrictions on publicly sharing data—e.g. participant privacy or use of data from a third party—those must be specified.

Reviewer #1: Yes

Reviewer #2: Yes

4. Is the manuscript presented in an intelligible fashion and written in standard English?

PLOS ONE does not copyedit accepted manuscripts, so the language in submitted articles must be clear, correct, and unambiguous. Any typographical or grammatical errors should be corrected at revision, so please note any specific errors here.

Reviewer #1: Yes

Reviewer #2: Yes

5. Review Comments to the Author

Please use the space provided to explain your answers to the questions above. You may also include additional comments for the author, including concerns about dual publication, research ethics, or publication ethics. (Please upload your review as an attachment if it exceeds 20,000 characters)

Reviewer #1: This paper addresses important, interesting, and timely questions about fishery management, and this reviewer finds the methods, at a fundamental level, appropriate and well implemented. My serious concerns are about the presentation and interpretation of the results. The authors “tie themselves into knots” by simulating myriads of possibilities and contorting results though scaling, weighting, and intractable/implicit aggregation. Due to all the transformations, I suspect the authors---like anyone reading the paper would---lost track of the correct interpretation of the results. One grave example: Scenario s5 is identified as achieving “the highest overall performance” and “but showed limited improvements in spawning biomass (Figure 6)” (Line 325). As far as this reviewer can ‘track’ the method to this result, it is technically wrong and leads to blatantly wrong/misguided interpretations. IF s5 scores “0.00” for biomass (B) (Table 5 and Figure 6) on a rescaled 0-1 scale, then the proper interpretation of zero is closer to total resource destruction than “limited improvements”!

The authors need to revisit how and what results they generate with a strong focus on simplicity, clarity, and tractability (for the reader). Simulation models provide empirically-guided conceptual results, hence the results presented do not need to cover all possible scenarios but should focus on those limited number of scenarios or permutations that produce interesting insights for fishery management! Much less, would be much more! The interpretation of those result needs to be conditioned by the assumptions/transformations along the way.

Response: We thank the reviewer for recognizing the importance of the question and the appropriateness of the underlying modeling framework. We also appreciate the reviewer’s strong concern about the original presentation and interpretation of the results. We agree that the previous version placed too much emphasis on transformed, scaled, and aggregated performance scores, which made the results difficult to interpret and led to overstatement of some scenario-level conclusions.

In response, we have substantially revised the Results and Discussion to emphasize transparent tradeoffs rather than a single overall ranking. Specifically, we have:

1. reorganized the original 16 scenarios into clearly defined scenario families: system-wide effort reduction, recreational effort reduction, recreational effort reduction with hypothetical full retention, fixed spatial closures, and dynamic spatial management;

2. revised the Results to focus on biological, fishery, and economic tradeoffs among scenario families rather than identifying a single “best” scenario;

3. removed the previous interpretation that scenario s5 achieved the “highest overall performance” and no longer describe any scenario as best overall;

4. replaced the previous primary emphasis on the 0–1 rescaled aggregate score with figures showing actual percent changes in biological/fishery metrics and actual economic changes in 2023 U.S. dollars;

5. moved aggregate scoring to an illustrative sensitivity analysis of management weighting assumptions;

6. added clearer figure captions and text explaining that percent changes are shown directly that dead discard reductions have the opposite management interpretation from landings or biomass increases, and that capped color scales are used only for visualization;

7. revised the Discussion to state that scenario rankings depend on management priorities and weighting assumptions, not on inherent properties of the model outputs.

We agree with the reviewer’s specific point regarding scenario s5. In the original manuscript, describing s5 as having “limited improvements in spawning biomass” was incorrect and misleading. In the revised manuscript, we now state that s5 increased recreational landings but produced a negative mean spawning biomass response and reduced spawning biomass for some species, including red snapper.

We have retained the full scenario set because it allows comparison among temporal, spatial, and spatiotemporal input-control mechanisms, but we now present the scenarios as structured management experiments rather than as a large set of competing policy recommendations. We believe this revision better reflects the conceptual role of the simulation model: to reveal tradeoffs among management mechanisms under specified assumptions, not to identify a single optimal policy.

More detailed comments in no particular order:

- Com_net metric represents gross ex-vessel revenue rather than net revenue; that is disingenuous. More materially, Com_net is probably redundant (as Figure 11 also shows), as Recreational Consumer Surplus is (only) comparable w

Attachment Submitted filename: PlosONE_response_letter.docx

# Peer review: Decision Letter 1

**DOI:** 10.1371/journal.pone.0355048.r003

**Authors:** Abdul Azeez Pokkathappada

17 Jul 2026

Navigating Complexity: Evaluating Management Measures for Multispecies Snapper-Grouper Fisheries in the Southeast U.S. Atlantic

PONE-D-26-09475R1

Dear Dr. Cao,

We’re pleased to inform you that your manuscript has been judged scientifically suitable for publication and will be formally accepted for publication once it meets all outstanding technical requirements.

Within one week, you’ll receive an e-mail detailing the required amendments. When these have been addressed, you’ll receive a formal acceptance letter and your manuscript will be scheduled for publication.

An invoice will be generated when your article is formally accepted. Please note, if your institution has a publishing partnership with PLOS and your article meets the relevant criteria, all or part of your publication costs will be covered. Please make sure your user information is up-to-date by logging into Editorial Manager at Editorial Manager® and clicking the ‘Update My Information' link at the top of the page. For questions related to billing, please contact billing support.

If your institution or institutions have a press office, please notify them about your upcoming paper to help maximize its impact. If they’ll be preparing press materials, please inform our press team as soon as possible -- no later than 48 hours after receiving the formal acceptance. Your manuscript will remain under strict press embargo until 2 pm Eastern Time on the date of publication. For more information, please contact onepress@plos.org.

Kind regards,

Abdul Azeez Pokkathappada, Ph.D.

Academic Editor

PLOS One

Additional Editor Comments (optional):

Kindly complete the minor correction suggested by reviewer.

Reviewers' comments:

Reviewer's Responses to Questions

**Comments to the Author**

Reviewer #1: (No Response)

Reviewer #2: All comments have been addressed

**********

2. Is the manuscript technically sound, and do the data support the conclusions?

Reviewer #1: Yes

Reviewer #2: Partly

**********

3. Has the statistical analysis been performed appropriately and rigorously?

Reviewer #1: Yes

Reviewer #2: Yes

**********

4. Have the authors made all data underlying the findings in their manuscript fully available?

The PLOS Data policy

Reviewer #1: Yes

Reviewer #2: Yes

**********

5. Is the manuscript presented in an intelligible fashion and written in standard English?

Reviewer #1: Yes

Reviewer #2: Yes

**********

Reviewer #1: The authors have addressed many of my original concerns. But the one central one is still not addressed: The presentation of too many results, with varying graphical tools and organization, requires too much of any reader. There are still 16 scenarios evaluated, and then 3 sensitivity analyses and both approaches serve the same purpose.

The 16 scenarios provide results for 9 measures (5 bio, 4 econ) across 6 species and overall. That’s 1008 data points for the reader to comprehend. That probably is already too much. For instance, Figure 6, which breaks out economic measures at the species level, gets just 2 sentences in the text (380-384). The sensitivity (to effort changes) under 3 differing assumptions (rec effort only-no-full retention; rec effort only-full retention; rec and com effort-full retention) provides results over continuous effort (10%-90%) for the 9 measures across 6 species. Hence the reader needs to comprehend 162 continuous lines…

Worse, the authors inexplicably switch the method of presentation. Fig 3, 4, 5, and 6 all present the 16 scenarios across the various measures and species. WHY is Figure 5 a dot-like plot when all the other figures are amounts (% or $, I think) with consistent color coding? Absolute WORST: Fig 5 reverses the order that the scenarios!!! Figure 5 needs to follow the same pattern as the other figures! [Aside, Figure 5 also clearly shows why including both commercial revenue and producer surplus is redundant: The dot cloud is exactly the same---just rescaled. Hence, I had recommended to just use one metric, also to limit the workload for the reader.]

Figures 7, 8, and 9 took me a long while to decipher: My major recommendation is “transposing” the figure and reordering the column panels. Logically, for left to right readers, the ‘least treatment’ scenario is the currently third one on the right (rec sector only, NO full retention). It really should be the first/left column/scenario and, internally, it should start with ‘100% effort remaining’ on the left or, better, 0% reduction, 10% reduction, 20% reduction. Notice that currently all lines converge on 0 all the way on the right of the figure! That should be the starting point. The second column should be ‘rec sector only, FULL retention, as it is most comparable to ‘rec sector only, NO full retention’. The third column should be ‘Both sectors, FULL’. So going from one column to the next adds treatments incrementally. All x-axis should go from 100% effort remaining to 10%, i.e., more intense treatment, or departure from the status quo, as the lines go to the right.

It looks like the two FULL retention scenarios (current middle and right side panels) compare the impact of the effort reduction to the global status quo/baseline. Hence at 90% Effort the lines appear far from “0” to which they should converge! Hence these are not really effort sensitivity curves at all---they are effort sensitivity AND the shift by going to full retention. Should these measures not be the percentage change from 100% Effort UNDER FULL RETETNION?

All this is why I think so many of the results are so redundant. Scenarios 1-6 look at various effort reduction and catch retention combinations---at 25% and 75% for both sectors or rec sector with or without full retention. Then the sensitivity analysis does it all again (always for 9 measures and 6 species). You could add another scenario at 50% effort and drop the sensitivity analyses?

Ideally, the authors would reduce the amount of results presented. At the very least, they need to make the presentation of their results consistent and intuitive to help the reader process so much data.

Reviewer #2: This revision represents a huge improvement!

The technical details (e.g., equations) about how fishing effort is redistributed under the various scenarios in the regional and depth closure scenarios remain unclear and likely unrealistic. I am willing to live with unrealistic, but increased specificity is important.

In the figures, it surprised me that red colors indicated increases and blue decreases. Consider reversing the color scale.

**********

what does this mean?). If published, this will include your full peer review and any attached files.

If you choose “no”, your identity will remain anonymous but your review may still be made public.

**Do you want your identity to be public for this peer review?**  For information about this choice, including consent withdrawal, please see our Privacy Policy

Reviewer #1: No

Reviewer #2: No

**********

# Editor report: Acceptance letter

**DOI:** 10.1371/journal.pone.0355048.r004

**Authors:** Abdul Azeez Pokkathappada

PONE-D-26-09475R1

PLOS One

Dear Dr. Cao,

I'm pleased to inform you that your manuscript has been deemed suitable for publication in PLOS One. Congratulations! Your manuscript is now being handed over to our production team.

At this stage, our production department will prepare your paper for publication. This includes ensuring the following:

* All references, tables, and figures are properly cited

* All relevant supporting information is included in the manuscript submission,

* There are no issues that prevent the paper from being properly typeset

You will receive further instructions from the production team, including instructions on how to review your proof when it is ready. Please keep in mind that we are working through a large volume of accepted articles, so please give us a few days to review your paper and let you know the next and final steps.

Lastly, if your institution or institutions have a press office, please let them know about your upcoming paper now to help maximize its impact. If they'll be preparing press materials, please inform our press team within the next 48 hours. Your manuscript will remain under strict press embargo until 2 pm Eastern Time on the date of publication. For more information, please contact onepress@plos.org.

You will receive an invoice from PLOS for your publication fee after your manuscript has reached the completed accept phase. If you receive an email requesting payment before acceptance or for any other service, this may be a phishing scheme. Learn how to identify phishing emails and protect your accounts at https://explore.plos.org/phishing.

If we can help with anything else, please email us at customercare@plos.org.

Thank you for submitting your work to PLOS ONE and supporting open access.

Kind regards,

PLOS ONE Editorial Office Staff

on behalf of

Dr. Abdul Azeez Pokkathappada

Academic Editor

PLOS One
